# *Eimeria bovis*-infected host cell-bystander cell interactions are essential for effective macromeront formation

**DOI:** 10.3389/fcimb.2026.1915455

**Published:** 2026-08-18

**Authors:** Jobst Fischer, Lisbeth Rojas-Barón, Carlos Hermosilla, Anja Taubert

**Affiliations:** Institute of Parasitology, Biomedical Research Center Seltersberg, Justus Liebig University Giessen, Giessen, Germany

**Keywords:** apicomplexan, *Eimeria bovis*, endothelial cells, gap junctions, mitochondrial transfer, parasite, parasite host-cell interactions, tunneling nanotubes

## Abstract

*Eimeria bovis* is an obligate intracellular apicomplexan parasite and the major agent of bovine coccidiosis. Intracellular *E. bovis* first merogony results in the formation of large macromeronts (≤ 400 µm) containing up to 140,000 merozoites I. This extensive replication imposes substantial metabolic stress on bovine endothelial host cells, leading to mitochondrial dysfunction and premature senescence. During macromeront development, non-infected bystander cells (BCs) accumulate around infected host cells and previous findings provided evidence for tunneling nanotube (TNT)-based interactions between infected cells and BCs. In the current study, we aimed to further analyze interactions between infected cells and BCs, potentially forming part of a multicellular support mechanism promoting macromeront development. To dissect BC-based reactions, we used a meront-transfer-system, which allowed for selective treatments of non-infected cells, serving as BCs in mixed cultures with *E. bovis* meront-carrying host cells (MCHCs). When addressing gap junction formation, protein expression profiles of *E. bovis*-infected cell layers revealed a significant upregulation of the connexins Cx37 and Cx40, but not of Cx43 during macromeront formation. In addition, *E. bovis* infection altered the subcellular distribution of Cx37, Cx40 and Cx43. The functional relevance of connexins was supported by blocking gap junction formation in BCs with carbenoxolone or Gap26 (inhibits Cx37, Cx40 and Cx43), both of which impaired parasite development. Besides connexins, we also studied TNT-based BC-MCHC-interactions in more detail. When quantitatively estimating BC-mediated donation of mitochondria to *E. bovis*-infected cells to improve MCHC bioenergetics, we showed that a high proportion of MCHCs (89%) obtained mitochondria from BCs. In line, the disruption of microtubule integrity by nocodazole treatments in BCs diminished both TNT formation and merozoite I production. Moreover, inhibitor-based impairment of BC metabolism demonstrated that intact glycolytic and glutaminolytic activities in addition to functional cholesterol esterification and trafficking are required in BCs to sustain effective merozoite I production in BC-MCHC-cultures. Collectively, the current findings support the hypothesis that effective intracellular *E. bovis* macromeront formation depends on an environmental BC-based support network involving BC metabolic integrity, short-range gap-junctional small molecule transfer, and long-range TNT-mediated mitochondria donation.

## Introduction

*Eimeria bovis* is a globally distributed, obligate intracellular apicomplexan parasite of cattle (*Bos* spp.) and water buffaloes (*Bubalus bubalis*) (Daugschies and Najdrowski, 2005; Dubey, 2018). Bovine eimeriosis, also known as coccidiosis, often remains subclinical but can also lead to severe hemorrhagic typhlocolitis in calves. Both scenarios result in substantial economic losses (Daugschies and Najdrowski, 2005). Accordingly, statistical surveys estimated *Eimeria* spp.-associated losses at 8-9% of the annual revenue, even when only considering clinical cases (Lassen and Ostergaard, 2012). At present, therapeutic options are restricted to a small number of metaphylactic drugs, and drug resistance has already been reported for ovine *Eimeria* spp (Odden et al., 2018).

Within the host, *E. bovis* undergoes successive phases of asexual replication (merogony I and II) followed by sexual development (gamogony). During first merogony, *E. bovis* proliferates in lymphatic endothelial cells of the intestinal villi. Unlike most other *Eimeria* species, *E. bovis* merogony I leads to the formation of exceptionally large intracellular macromeronts, reaching sizes of up to 400 µm and containing more than 140,000 merozoites I per meront (Hammond et al., 1946, Hammond et al., 1966). This extensive and long-lasting intracellular proliferation (~ 3 weeks) requires high amounts of energy and structural components, prompting *E. bovis* to profoundly manipulate host cell functions to meet its metabolic and structural demands (Hermosilla et al., 2008; Taubert et al., 2010). Consequently, a plethora of host cell pathways related to lipid/cholesterol metabolism, glycolysis, innate immune responses, apoptosis, and cytoskeletal organization are markedly altered during *E. bovis* first merogony (Taubert et al., 2006; Hermosilla et al., 2008; Hamid et al., 2014, Hamid et al., 2015; Taubert et al., 2018; Velásquez et al., 2021b; Silva et al., 2022). In addition, *E. bovis* infection induces host cell cycle arrest at G_1_ phase and triggers host cellular mitochondrial dysfunction and premature senescence (Velásquez et al., 2021a), collectively indicating a severe parasite-driven disruption of host cell metabolism and energy homeostasis. Despite these extreme cellular stresses, macromeront-carrying host cells (MCHCs) remain viable and allow for long-lasting parasite development until merozoite I release (Hermosilla et al., 2012). The discrepancy between severe host cell impairment and sustained parasite proliferation raised the question of how the functional competence of *E. bovis*-infected host cells is maintained throughout the long-lasting macromeront development (~ 3 weeks). Previous work showed that non-infected bystander cells (BCs) accumulate around MCHCs and may form part of a multicellular support system (Fischer et al., 2025). Moreover, *E. bovis* infection upregulated the formation of tunneling nanotubes (TNTs) (Fischer et al., 2025), which allow for the intercellular exchange of organelles like mitochondria (Cervantes and Zurzolo, 2021). In other contexts, TNT-mediated healthy mitochondria transfer enabled their incorporation into the endogenous network of impaired recipient cells, thereby allowing these cells to recover and to return to a normal operating state (Liu et al., 2014; Luchetti et al., 2022). During pathogen infections, TNTs are a well-documented tool for efficient bacterial and viral spread to spatially separated uninfected cells (Kim et al., 2019; Jansens et al., 2020; Jahnke et al., 2022; Lv et al., 2024). Referring to parasites, only one study reports that *Leishmania donovani* induces TNT formation in B cells and uses these structures to disseminate among cells (Stögerer et al., 2023). Notably, the utilization of TNTs to improve bioenergetics of potentially exhausted parasite-infected host cells has not yet been addressed. However, we recently illustrated that mitochondria are indeed transferred from BCs to *E. bovis*-infected cells and vice versa (Fischer et al., 2025), in the latter case potentially functioning to discard dysfunctional mitochondria, which augment at late phase of infection (Velásquez et al., 2021b).

Given that BCs are in close contact with *E. bovis*-infected host cells, communication mechanisms other than TNTs may also contribute to macromeront sustainment. Gap junctions may represent one such mechanism. Gap junctions are transmembrane structures formed between adjacent cells by the docking of two hemichannels, each composed of six connexin (Cx) subunits, enabling direct intercellular transfer of small molecules (e. g. metabolites like ATP, ions) via cytoplasmic continuity (Evans and Martin, 2002). In a resting state, these hemichannels are typically closed, but adapt to specific stimuli like cell stress by increasing their open probability (Johansen et al., 2011). In general, mammalian endothelial cells predominantly express Cx37, Cx40 and Cx43 (Van Rijen et al., 1997). Notably, Cx43 has also been shown to be directly involved in the signaling pathway promoting TNT formation (Tishchenko et al., 2020). So far, the role of connexins has scarcely been studied in coccidia-infected host cells. Connexins are considered as vertebrate proteins and do not seem to be encoded by apicomplexan parasites (Wastling et al., 2009; Vega et al., 2013). Nevertheless, a former study reported that *T. gondii* infections caused gap junction disappearance in brain cells even though not affecting expression levels of Cx26 and Cx43 (Campos de Carvalho et al., 1998).

The aim of the current work was to broaden the current knowledge on the relevance and mechanisms of bystander cell-promoted aid for effective *E. bovis* macromeront formation by studying the role of endothelial connexins, metabolic- and cytoskeletal requirements in BCs, and further analyzing TNT-mediated mitochondria transfer between BCs and MCHCs. Throughout this study, the term bystander cells (BCs) refers to non-infected BUVECs associated with infected host cells in *E. bovis*-infected cultures. Depending on the experimental context, BCs comprise non-infected cells accumulating directly around or located adjacent to MCHCs (i.e. nearby), or the non-infected recipient BUVEC layer used in meront-transfer experiments. Beyond *E. bovis*, our findings will provide new insight into environmental multicellular support mechanisms which may also be key for further macromeront-forming *Eimeria* species or other intracellular apicomplexan parasites.

## Materials and methods

### Eimeria bovis experimental animal infection

Two-week-old male Holstein Friesian calves (*n* = 3) were purchased from a local dairy farm and maintained under parasite-free conditions in stainless-steel metabolic cages (Woetho, Emmendingen, Germany) within an experimental large animal facility at the Institute of Parasitology, Justus Liebig University Giessen, equipped with an airlock entrance. Animals were routinely screened for parasitic infections at three-day intervals. Calves were fed a milk replacer (Milkvit, Trouw Nutrition Deutschland GmbH, Burgheim, Germany) and a commercial concentrate (Raiffeisen Vital eG, Hamm, Germany). Water and sterilized hay were provided *ad libitum*. At 7–8 weeks of age, calves were orally infected with 3 × 10³ sporulated *E. bovis* (strain H) oocysts, which had been washed three times in water by centrifugation (600 × g, 15 min) prior to infection (Silva et al., 2022). The *E. bovis* strain H used in this study was originally isolated from field cases in northern Germany and has since been maintained by serial passages in parasite-free male Holstein Friesian calves (Fiege et al., 1992). The animal health status was routinely monitored throughout the entire infection period. All experimental procedures were performed in accordance with the guidelines of the Justus Liebig University Giessen Animal Care Committee and were approved by the Ethics Commission for Experimental Animal Studies of the State of Hesse (Regierungspräsidium Giessen; GI 18/10 No. V1/2022; JLU-No. 0001-V), in compliance with current German animal protection legislation.

### *Eimeria bovis* oocyst isolation

Calves were orally infected as described above. Subsequently, oocyst collection, sporulation, and storage were performed according to previously published studies (Taubert et al., 2018). For excystation, sporulated oocysts were suspended in a sterile aqueous solution containing 0.022 M _L_-cysteine hydrochloride (Sigma-Aldrich, St. Louis, MO, USA) and 0.2 M NaHCO_3_ (Sigma-Aldrich), followed by incubation for 16–20 h at 37 °C under a 100% CO_2_ atmosphere. After incubation, oocysts were pelleted (600 × g,15 min, 20 °C) and resuspended in 1x Hank’s balanced salt solution (HBSS; Gibco, Fisher Scientific GmbH, Schwerte, Germany) containing 0.04% (w/v) trypsin (Sigma-Aldrich) and 8% (v/v) sterile-filtered (0.2 µm filter; Sarstedt, Nümbrecht, Germany) bovine bile obtained from a local abattoir. Oocysts were incubated for up to 4 h (37 °C, 5% CO_2_) under continuous microscopic monitoring. Released sporozoites were washed twice (600 × g, 15 min, 20 °C) in sterile medium (M199; Sigma Aldrich), passed through a 10 µm pore-size filter (pluriStrainer, PluriSelect, Leipzig, Germany, Life Science) according to López-Osorio et al. (2020), and counted in a Neubauer chamber (Karl Hecht GmbH & Co KG, Sondheim vor der Rhön, Germany). Purified *E. bovis* sporozoites were subsequently used for infection of primary bovine umbilical vein endothelial cells (BUVEC).

### Isolation and maintenance of primary BUVEC

Primary BUVEC were isolated from bovine umbilical cords following established protocols by Taubert et al. (2018). Briefly, umbilical cords were collected aseptically from calves delivered by caesarean section. Endothelial cells were obtained by treatments with 0.025% collagenase type II (Worthington Biochemical Corporation, Lakewood, NJ, USA) suspended in aqueous Pucks solution [NaCl (0.8% w/v), KH_2_PO_4_ (0.015% w/v), KCl (0.04% w/v), CaCl_2_ (0.0012% w/v), all Carl Roth GmbH & Co. KG, Karlsruhe, Germany, MgSO_4_ x 7 H_2_O (0.0152% w/v), NaH_2_PO_4_ (0.039% w/v) Sigma-Aldrich]. The collagenase solution was infused into the lumen of ligated umbilical veins. Following an incubation period of 20 min (37 °C, 5% CO_2_), the cell suspension was collected in cell culture medium (20 ml) and supplemented with 1 ml fetal calf serum (FCS, Gibco) to inactivate collagenase. After washing (350 × g,12 min, 20 °C), cells were resuspended in complete endothelial cell growth medium (ECGM, PromoCell, Heidelberg, Germany, supplemented with 5% FCS), seeded in 75-cm^2^ tissue plastic culture flasks (Greiner Bio-One GmbH, Frickenhausen, Germany) and incubated at 37 °C in 5% CO_2_ atmosphere. BUVECs were continuously cultured in modified ECGM (modECGM) medium [EGCM diluted at 30% in M199 medium, supplemented with 5% FCS (Gibco), 1% penicillin and streptomycin (Sigma-Aldrich)]. Medium was exchanged every second to third day, and BUVECs of passages 1–4 were used for infection experiments.

### Enrichment of *Eimeria bovis* macromeront-carrying host cells

BUVECs (*n* = 4) were seeded into 75-cm² tissue culture flasks (Greiner Bio-One GmbH) coated with bovine fibronectin (1:200 in PBS, Sigma Aldrich, F1141-2MG, St. Louis, MO, USA). When reaching 90% confluency, cells were infected with *E. bovis* sporozoites (7.5 × 10^5^ - 1.5 × 10^6^ sporozoites/flask). Medium was replaced 24 h post infection (p. i.) and subsequently every two days. At day 14 p. i., infected BUVEC were detached using trypsin [0.25% (w/v), Sigma-Aldrich, 4 ml per 75-cm² tissue flasks, 5 min, 37 °C]. Enzymatic digestion was stopped by supplementation of 8 ml modECGM. The cell suspension was transferred to a 15 ml Falcon™ tube (Thermo Fisher Scientific, Waltham, MA, USA) and sedimented (400 × g, 5 min, RT). The cell pellet was thoroughly resuspended and incubated (15–45 min) in 5 ml accutase (Accutase Cell Detachment Solution ACC-1B, Capricorn Scientific, Ebsdorfergrund, Germany) to obtain a single-cell suspension. Cell dissociation was monitored microscopically every 10 min. After complete dissociation into a single-cell suspension, the cells were filtered by a sterile 20 μm cell strainer (pluriStrainer, PluriSelect, Leipzig, Germany) mounted on an adaptor (pluriSelect, pluriStrainer, PluriSelect) and placed on a 50 ml Falcon™ tube to remove non-infected BUVEC (which passed through) to enrich MCHCs (> 20 μm diameter), which remained on top. After extensive washing with modECGM, the strainer was inverted and MCHCs were flushed out with 6 ml modECGM into a 6-well culture plate (Sarstedt AG & Co. KG, Nürnbrecht, Germany). MCHC enrichment was verified microscopically. The filtration procedure was repeated once or twice until a purity ratio of MCHCs to non-infected BUVEC ≥ 1 was achieved.

### Protein extraction for western blotting

*E. bovis*-infected and control BUVEC were trypsinized (2 ml trypsin/T25 flask, 5 min, 37 °C) at different time points of infection (e. g. 4, 8, 12, 18, 22 days p. i.) and pelleted (500 x g, 5 min). The cell pellet was washed with 1 ml of 1X PBS and centrifuged (500 x g, 12 min). Cells were resuspended in radioimmunoprecipitation assay (RIPA) buffer [50 mM Tris-HCl, 1% NP-40, 0.5% sodium-deoxycholate, 0.1% sodium dodecyl sulphate (SDS), 150 mM NaCl, 2 mM ethylenediaminetetraacetic acid (EDTA), 50 mM sodium fluoride in aqua dest., all Carl Roth GmbH + Co. KG, Karlsruhe, Germany] supplemented with protease inhibitors (200X, P8849, Sigma-Aldrich), 200 mM phenylmethylsulfonyl fluoride (PMSF, AV141032, Abcam, Cambridge, UK) and 100 mM sodium orthovanadate (Na_3_VO_4_, AB120386, Abcam). Cells were sonicated (20 s, five times in freezing water) and centrifuged (1 x 10^4^ g, 10 min, 4 °C). Supernatants were further processed using a Pierce™ Protein Assay Kit (23225 Thermo Scientific) following the company’s instructions. The protein content of the samples was estimated by measurements at 562 nm in a Varioskan Flash Multimode Reader (Thermo Fisher Scientific).

### SDS-PAGE and immunoblotting

Protein samples were supplemented with Laemmli-β-mercaptoethanol loading buffer (1610747, Bio-Rad Laboratories, Hercules, CA, USA). After heating (95 °C) for 5 min, protein samples (40 μg protein/slot) were loaded on 10 - 15% polyacrylamide gels and separated by electrophoresis (50 V/15 min + 300 V/45 min, Mini-PROTEAN^®^ Tetra Cell, Bio-Rad Laboratories, Hercules, CA, USA). Proteins were then transferred to polyvinylidene difluoride membranes (7 min, 2.5A, 25 V, Trans-Blot Turbo Transfer System), blocked in 3% bovine serum albumin (BSA) diluted in Tris-buffered saline (TBS) [50 mM Tris-HCl, pH 7.6; 150 mM NaCl; 0.1% TWEEN20 (P1379, Sigma-Aldrich)] for 1 h at RT and then reacted with primary antibodies (**Table 1**) diluted in blocking solution (overnight, 4 °C). Following three washings with TBS-TWEEN20 (0.1%) buffer, blots were incubated in corresponding secondary antibodies (**Table 1**) diluted in blocking solution (30 min, RT). After three washings with TBS-TWEEN20 (0.1%) buffer, immunoreactions were visualized by a chemiluminescence detection system (ECL Plus, Thermo Fisher Scientific) in a ChemoDoc MP Imager^®^ (Bio-Rad Laboratories). Protein sizes were estimated using a molecular weight ladder (PageRuler™ Prestained Protein Ladder ∼10–180 kDa, Thermo Fisher Scientific). Protein band intensities were quantified by ImageJ (imagej.net). Notably, connexins typically showed a multi-band pattern, most likely due to post-translational modifications (Musil et al., 1990; Guo et al., 2017). Therefore, all specific protein bands of expected molecular masses were included for quantification. Vinculin expression was used as internal loading control.

**Table 1:** Antibodies and dyes used in this study.

Antibodies / Dyes	Company	RRID	Cat. Number	Origin	Dilution
Primary antibodies					
Anti-Cx37	Invitrogen	AB_2533524	404200	Rabbit	1:85 (IF), 1:200 (WB)
Anti-Cx40	Invitrogen	AB_2533346	364900	Rabbit	1:85 (IF), 1:200 (WB)
Anti-Cx43	Cell Signaling	AB_2940791	83649	Rabbit	1:200 (IF), 1:500 (WB)
Secondary antibodies					
AlexaFluor 488	ThermoFisher	AB_143165	A11008	Goat	1:400
Dyes					
MitoView™ Green	Biotium™	–	70054	–	100 nM
DAPI Fluromount-G™	Invitrogen™	–	495952	–	1 drop / coverslip

### TNT quantification

Previous studies have shown that TNT formation can be altered by connexins, arachidonic acid, _N_-acetylcysteine or nocodazole treatments in different cell types (Astanina et al., 2015; Tishchenko et al., 2020; Lin et al., 2024; Needs et al., 2024; Zhao et al., 2024). To validate these findings for the current cell type and to analyze the effect of connexin inhibitors, BUVEC (3.5 x 10^5^/well, *n* = 3 - 7) were seeded on fibronectin-coated 12-well plates (Greiner Bio-One GmbH) in technical triplicate. One day after seeding, cells were treated with nocodazole (50 nM, M1404, Merck/Sigma-Aldrich), arachidonic acid (80 µM, 10931, Merck/Sigma-Aldrich), _N_-acetylcysteine (1 µM, A9165, Sigma-Aldrich), carbenoxolone (10 µM, J63714.03, Thermo Scientific), Gap19 (10 µM, S6946, Selleck Chemicals, Houston, TX, USA) or Gap26 (16 µM, HY-P1082, MedChemExpress, NJ, USA) for 24 h. These compound concentrations had previously been confirmed as non-toxic via cell viability tests (**Supplementary Figure 1**). After 48 h, subconfluent cell layers were used for TNT quantification according to Tishchenko et al. (2020). For each BUVEC isolate, microscopic images were randomly taken using a phase contrast microscope (100x-200x magnification) and analyzed for TNT formation by ImageJ software using Olympus Viewer and cell counter plugin. All membrane protrusions and connecting membrane bridges with diameters ranging from 70–4000 nm were counted.

### Inhibition of gap junctions/connexins in bystander cells

**Figure 1:**
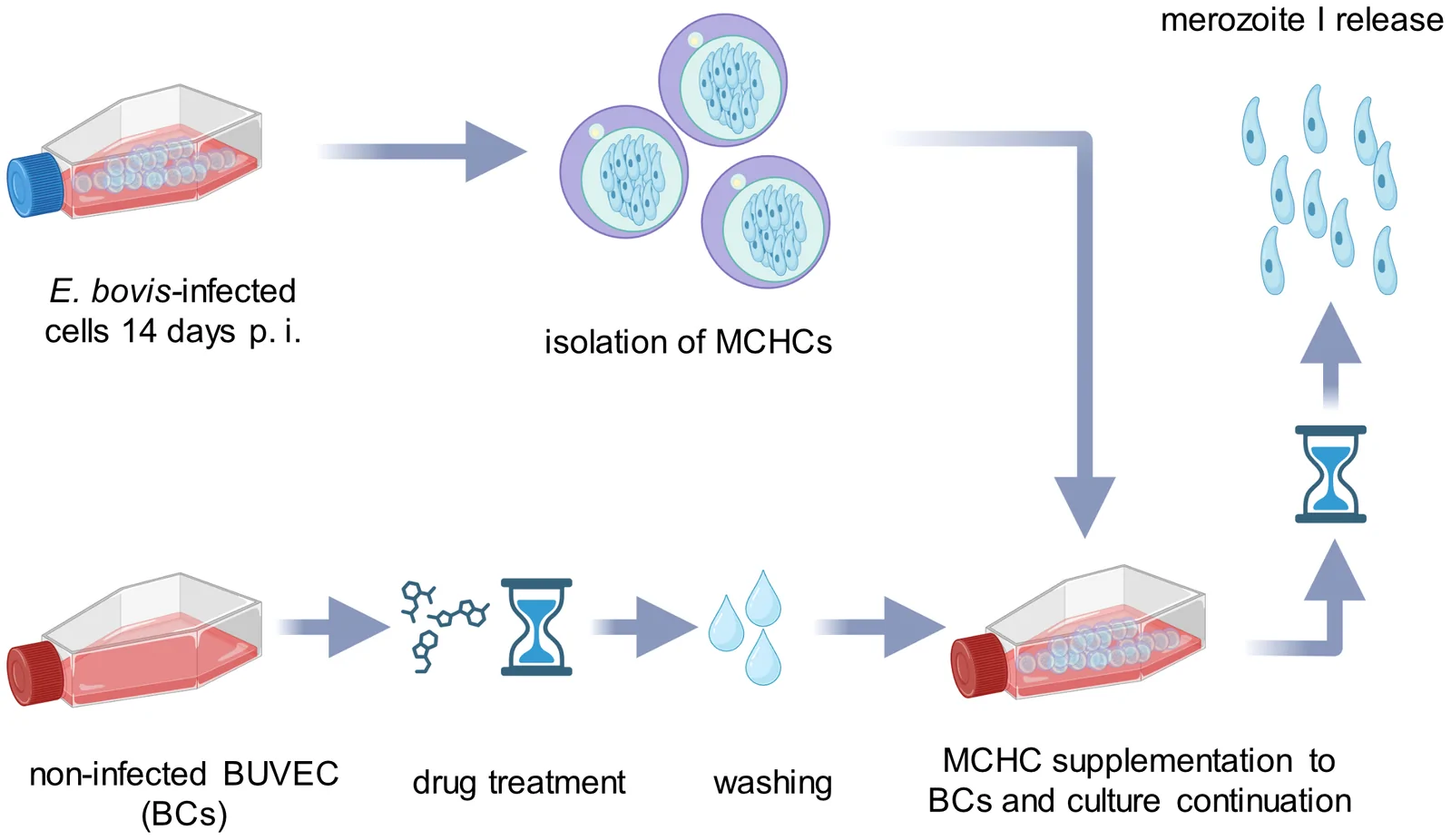
Workflow experimental set up to assess the effect of bystander cell treatments on *E. bovis* macromeront development. Created using BioRender.

To assess the relevance of BC-derived gap junction formation for *E. bovis* macromeront development, non-infected BUVEC (*n* = 4) were seeded on bovine fibronectin-coated 24-well plates (Greiner Bio-One GmbH) and treated for 24 h with Gap19 (10 µM), carbenoxolone (10 µM) or Gap 26 (16 µM) or left untreated in plain modECGM (control). After thorough washing in PBS and medium replacement, enriched MCHCs (14 days p. i.) were transferred to treated BUVEC layers (now functioning as BCs, **Figure 1**), allowed to integrate and to develop by culturing for further 13 days. To monitor parasite development, merozoites I released into the cell supernatants were collected and quantified in a Neubauer chamber from 1-day post transfer onwards.

### Inhibition of selected pathways in bystander cells

To assess metabolic and other requirements for BC-based support of *E. bovis* macromeront development, non-infected BUVEC (*n* = 4) were seeded on bovine fibronectin-coated 24-well plates (Greiner Bio-One GmbH) and treated with rotenone (Rot, 1 µM, AC132370050, Merck Millipore, Billerica, MA, USA), nocodazole (Noco, 50 nM, 358240100, Thermo Scientific), 6-Diazo-5-oxo-L-norleucine (DON, 2 µM, D2141, Sigma), arachidonic acid (AA, 80 µM, 181196, Merck/Sigma-Aldrich), _N_-acetylcysteine (NAC, 1 µM, A7250, Sigma-Aldrich), fluordeoxyglucose(^18^F) (FDG), 100 µM, 29702, Chemodex AG, St. Gallen, CH), ethylfluoracetate (EF, 1 mM, 163813, Sigma-Aldrich), U18666A (2 µM, HY-107433, MedChemExpress), CL976 (5 µM, C3743 Sigma-Aldrich), or left in plain modECGM (control). Adequate compound concentrations had previously been identified as non-toxic via cell viability tests (see **Supplementary Figure 1**). After thorough washing in PBS and medium replacement, enriched MCHCs (14 days p. i.) were supplemented to pre-treated BUVEC layers (now functioning as BCs, **Figure 1**), allowed to integrate and to develop by culturing for further 13–16 days. To assess inhibitor effects on parasite development, free-released merozoites I present in cell supernatants were quantified in a Neubauer chamber from 1-day post transfer onwards.

### Quantification of mitochondrial exchange between MCHCs and BCs

To quantify mitochondrial transfer from BC to MCHC, BUVECs functioning as recipient BCs were grown in a 12-well cell culture plate (Sarstedt AG & Co. KG), stained by MitoView™Green (100 nM, 70054, 30 min, Biotium, Fremont, CA, USA) and extensively washed (5x) with PBS to remove dye remnants. After the transfer of unstained MCHCs from 14 days p. i. to MitoView™Green-stained BUVEC, the MCHCs were controlled microscopically for fluorescence shortly after transfer (30–45 min) to exclude leakage-based dye carry-over. Thereafter, MCHCs were monitored for mitochondria transfer from BCs at 24 h post MCHC supplementation by estimating green signals in MCHCs, which derived from MitoView™Green-stained mitochondria being delivered by BCs. The fluorescence intensity of MCHCs compared to the donor BUVEC layer was quantified by ImageJ software directly post transfer (30–45 min) and 24 h later.

### Visualization of connexin and connexin plaque accumulation

To visualize connexins (Cx37, Cx40, Cx43) by immunofluorescence staining, BUVECs were grown on glass coverslips (⊘1.5 cm) deposited in a 12-well cell culture plate (Sarstedt AG & Co. KG), infected with *E. bovis* sporozoites (5 x 10^4^/well) and fixed with 4% paraformaldehyde (PFA) for 20 min at RT. After fixation, the cells were washed three times with PBS and blocked for 1 h (3% BSA and PBS) and then incubated in permeabilization solution (PBS with 3% BSA, 0.3% Triton X-100, Sigma Aldrich) for 15 min at RT. Thereafter, samples were incubated with primary antibodies (**Table 1**) diluted in PBS with 1% BSA and 0.5% Tween20 (Sigma-Aldrich) overnight at 4 °C in a humidified chamber. After three additional washes in PBS, the samples were incubated with secondary antibodies diluted in PBS with 3% BSA and 0.5% Tween 20 (**Table 1**) for 30 min at RT in darkness. Cell nuclei were stained while mounting the coverslips on glass slides with 4′, 6-diamidino-2-phenylindole (DAPI) present in mounting medium (495952, Fluoromount G, Invitrogen, Carlsbad, CA, USA). To assess subcellular connexin distribution during *E. bovis* infection rather than total fluorescence intensity, we established a semi-quantitative classification strategy. Individual BUVECs in non-infected BUVEC layers and infected BUVEC layers were categorized according to low (type 1), medium (type 2) and high (type 3) presence of cytoplasmic connexin clusters and/or membrane-associated plaques, thereby separately registering cell subpopulations of BCs and *E. bovis*-infected host cells (IHC). In detail, type 1 indicated low diffuse and/or perinuclear connexin presence, with no or few prominent accumulations (1–5 clusters), score 2 indicated moderate multifocal staining (6–20 clusters) and/or low membrane-associated signal (plaques), and score 3 indicated multifocal accumulation with > 20 clusters and/or prominent membrane-associated signal (plaques). The classification was performed at single-cell level using three biological replicates per condition. In total, ≈ 500 BUVEC for whole cell layers, ≈ 200 BCs and ≈ 70 IHCs were evaluated for each condition. Representative examples of the types are provided in **Supplementary Figure 3**.

### Image acquisition and analyses

For phase contrast and fluorescence analyses, samples were analyzed by an inverted microscope (IX81, Olympus, Olympus Plan NA 0.25/10x, NA 0.45/20x Shinjuku City Tokyo, Japan, or BZ-X800, Plan Fluorite NA 0.45/20x Keyence, Neu-Isenburg, Germany). Image analysis was carried out by ImageJ and the BZ-X800 Viewer software (Keyence). Confocal z-Stacks and images were acquired with a ReScan Confocal instrumentation (RCM 1.1 Visible, Confocal.nl) equipped with a fixed 50 µm pinhole size and combined with a Nikon Ti-2 Eclipse microscope (Plan Apo 1.4/60x1.5x) with a motorized Z-stage (DI1500, Nikon). The RCM unit was connected to a Toptica CLE laser with the following excitations: 405/488nm and operated by the NIS-Elements software. 3D rendering was performed in NIS-Elements software (volume viewer).

### Scanning electron microscopy

BUVEC infected with *E. bovis* sporozoites were seeded on 10 mm glass coverslips (Nunc™, Thermonax™, Thermo Scientific) coated with poly-l-lysine (Sigma-Aldrich). Cells were fixed in 2.5% glutaraldehyde (Merck), post-fixed in 1% osmium tetroxide (Merck), washed in distilled water, dehydrated, dried by CO_2_ treatment, and sputtered with gold. Samples were analyzed using a Philips XL30 scanning electron microscope (SEM; Institute of Anatomy and Cell Biology, Justus Liebig University Giessen, Germany).

### Statistical analysis

All data were expressed as arithmetic mean with standard deviation. Statistical comparisons between non-infected and infected, or non-treated and treated cells used Mann-Whitney-U-test or Welch-t-test. Whenever multiple comparison was necessary Kruskal-Wallis-Test was performed with correction of Benjamini and Yekutieli. Analysis was conducted using GraphPad Prism^®^10 or SPSS software applying a significance level of 5% and a false discovery rate of 5% (adjusted *p* value).

## Results

### *Eimeria bovis* infection upregulates Cx37- and Cx40 expression in host cell layers

**Figure 2:**
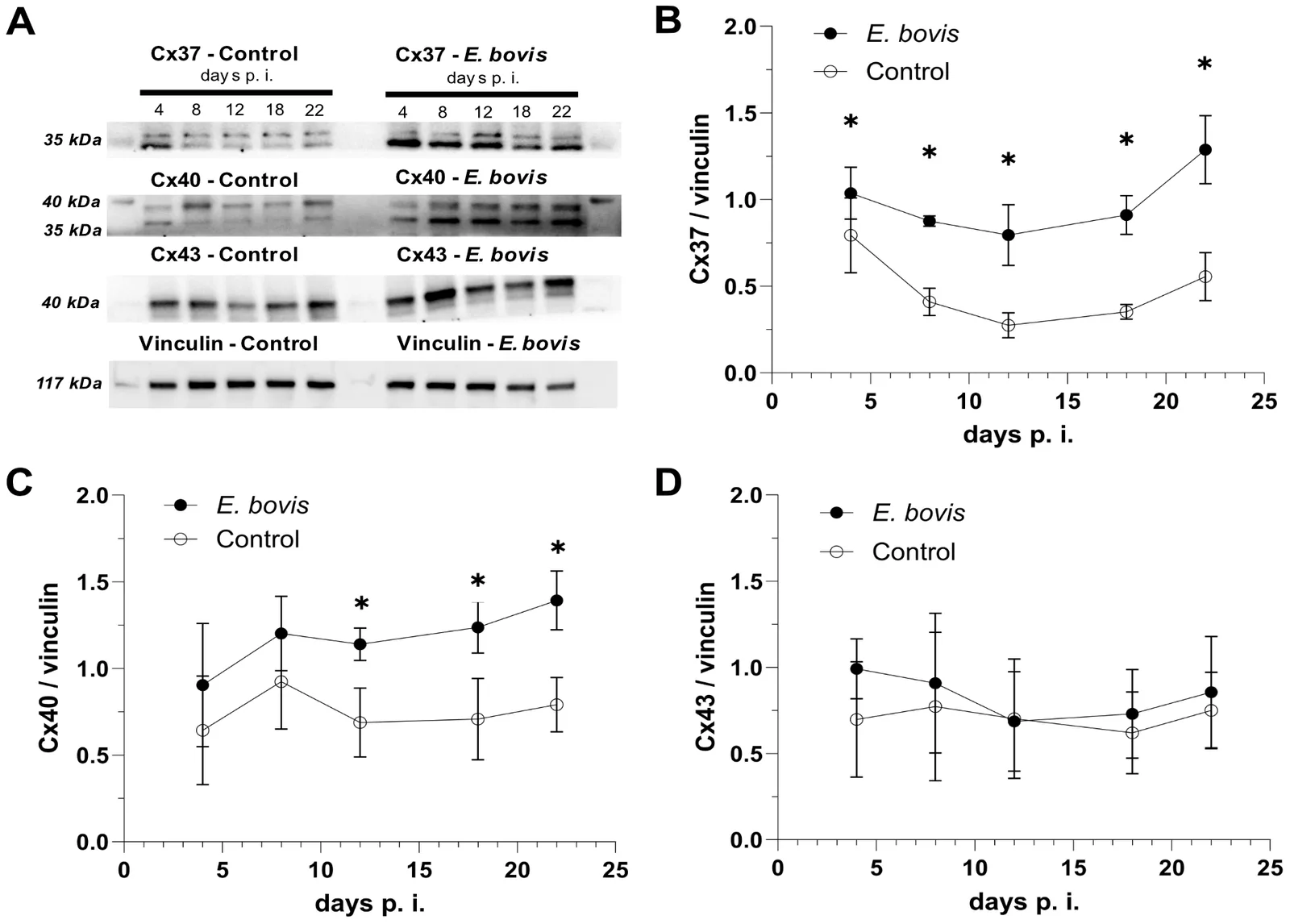
Expression profiles of Cx37, Cx40 and Cx43 in *E. bovis*-infected BUVEC. Illustration of exemplary Western blots on Cx37, Cx40, Cx43 and vinculin **(A)**, quantification of Cx37 **(B)**, Cx40 **(C)** and Cx43 **(D)** expression throughout *E. bovis* macromeront formation compared to non-infected control cells. Arithmetic mean and standard deviation of 3–4 biological replicates, adjusted *p ≤ 0.05.

Our recent study showed that MCHCs and BCs interact through TNTs and we hypothesized that BCs support MCHCs to improve their bioenergetics (Fischer et al., 2025). Given that efficient intercellular crosstalk is pivotal for the maintenance of cellular functions after stress events, both cell types need to communicate with each other. Besides TNTs, gap junction formation represents one mechanism of intercellular communication and potential support. Gap junctions are intercellular hemichannels composed of connexins, which allow for ionic shifts or small molecule transfer (e.g. metabolites, ATP) between adjacent cells (Evans and Martin, 2002). We therefore first examined whether *E. bovis* infection alters the expression of the main endothelial connexins Cx37, Cx40 and Cx43 throughout macromeront formation. Cx37 expression was significantly increased from day 4 p. i. onwards (at day 4 p. i.: adjusted *p*-value (*p) = 0.024, at 8, 12, 18 and 22 days p. i.: all *p = 0.008, **Figure 2B**). In addition, Cx40 expression was significantly enhanced by *E. bovis* infection from day 12 onwards (12, 18, 22 p. i.: all *p = 0.020, **Figure 2C**) whilst Cx43 expression was not changed significantly by infection (**Figure 2D**).

### *Eimeria bovis* infection induces spatial changes in connexin localization and enhances gap junction plaque formation in BUVEC layers

**Figure 3:**
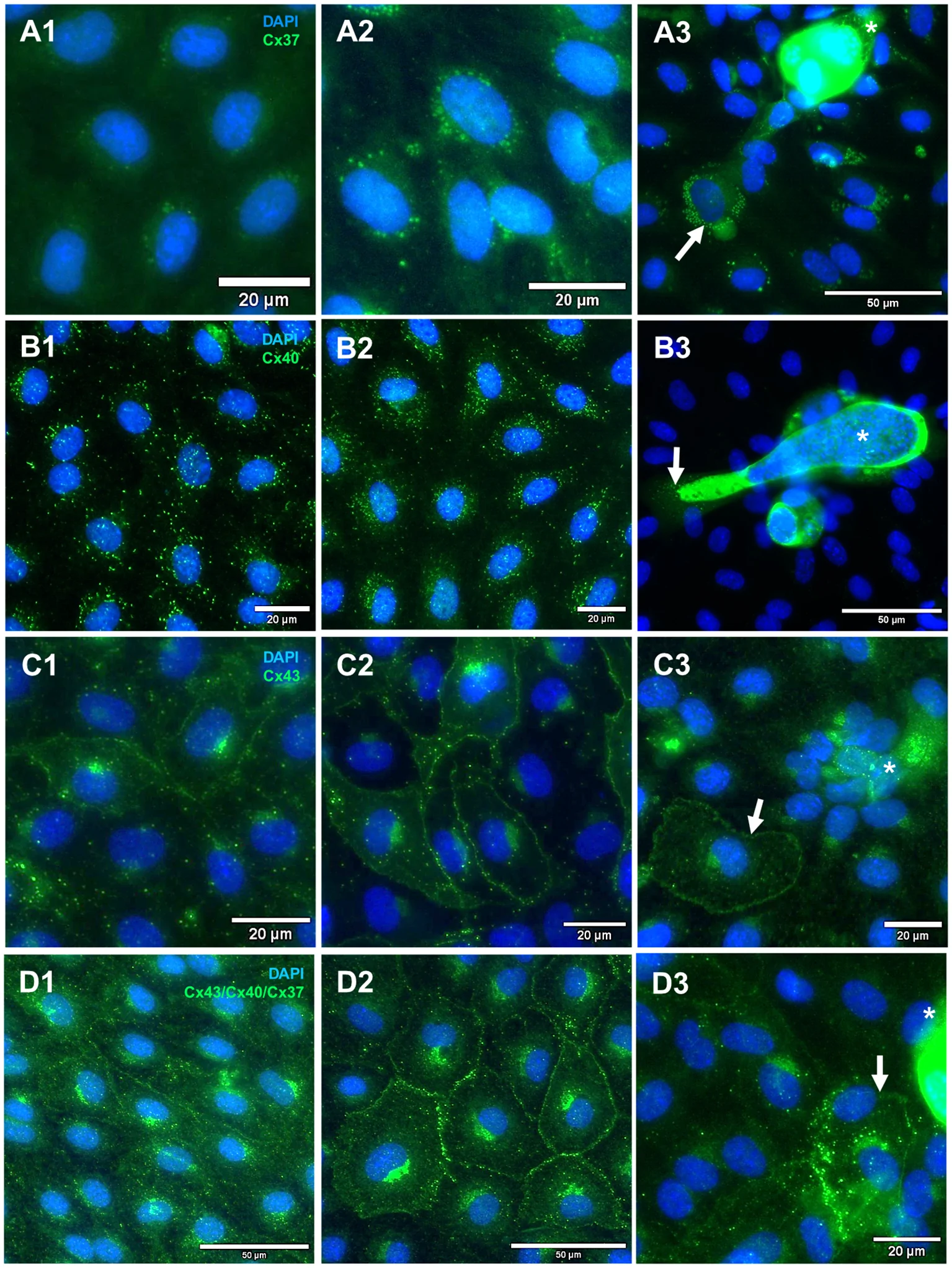
Immunofluorescence staining of connexins (Cx) in non-infected and *E. bovis*-infected BUVEC. **(A1)** Non-infected BUVEC showing diffuse Cx37 staining with few perinuclear Cx37-clusters. **(A2)** *E. bovis*-infected BUVEC layer containing non-infected BUVEC with increased numbers of perinuclear Cx37-clusters. **(A3)** *E. bovis*-infected BUVEC layer containing an MCHC (17 days p. i., asterisk) with an enhanced green signal, partially consistent with autofluorescence of the parasitophorous vacuole (PV). Adjacent cells also display an increased number of Cx37 clusters (white arrow). **(B1)** Non-infected BUVEC showing diffuse Cx40 staining with cytoplasmic Cx40 clusters. **(B2)** *E. bovis*-infected BUVEC layer with non-infected BUVEC showing a higher number of cytoplasmic Cx40 clusters. **(B3)** *E. bovis*-infected BUVEC layer containing MCHCs (17 days p. i., asterisk) with an intense green signal, partially consistent with PV autofluorescence. Cx40-positive clusters are also visible inside the cytoplasm of a MCHC (white arrow). **(C1)** Non-infected BUVEC showing diffuse Cx43 staining with perinuclear Cx43-positive clusters and membrane associated Cx43 plaques. **(C2)** *E. bovis*-infected BUVEC layer with non-infected BUVEC showing more prominent membrane associated Cx43 plaques. **(C3)** *E. bovis*-infected BUVEC layer showing a non-infected cell close to an infected cell, displaying a translocation of Cx43 to the cell membrane and cell membrane-associated Cx43-plaques (arrow); the MCHC (12 days p. i., asterisks) shows Cx43-positive clusters. **(D1)** Non-infected BUVEC with combinatory Cx37/Cx40/Cx43 staining showing membrane-associated and cytoplasmic (especially perinuclear) localization of connexins. **(D2)** *E. bovis*-infected BUVEC layer showing an increased translocation of connexins to the cell membrane, connexin plaque formation and perinuclear connexin clusters. **(D3)** *E. bovis*-infected BUVEC layer containing an MCHC (asterisk) and BC (arrow) both with enhanced connexin plaque and cluster formation.

**Figure 4:**
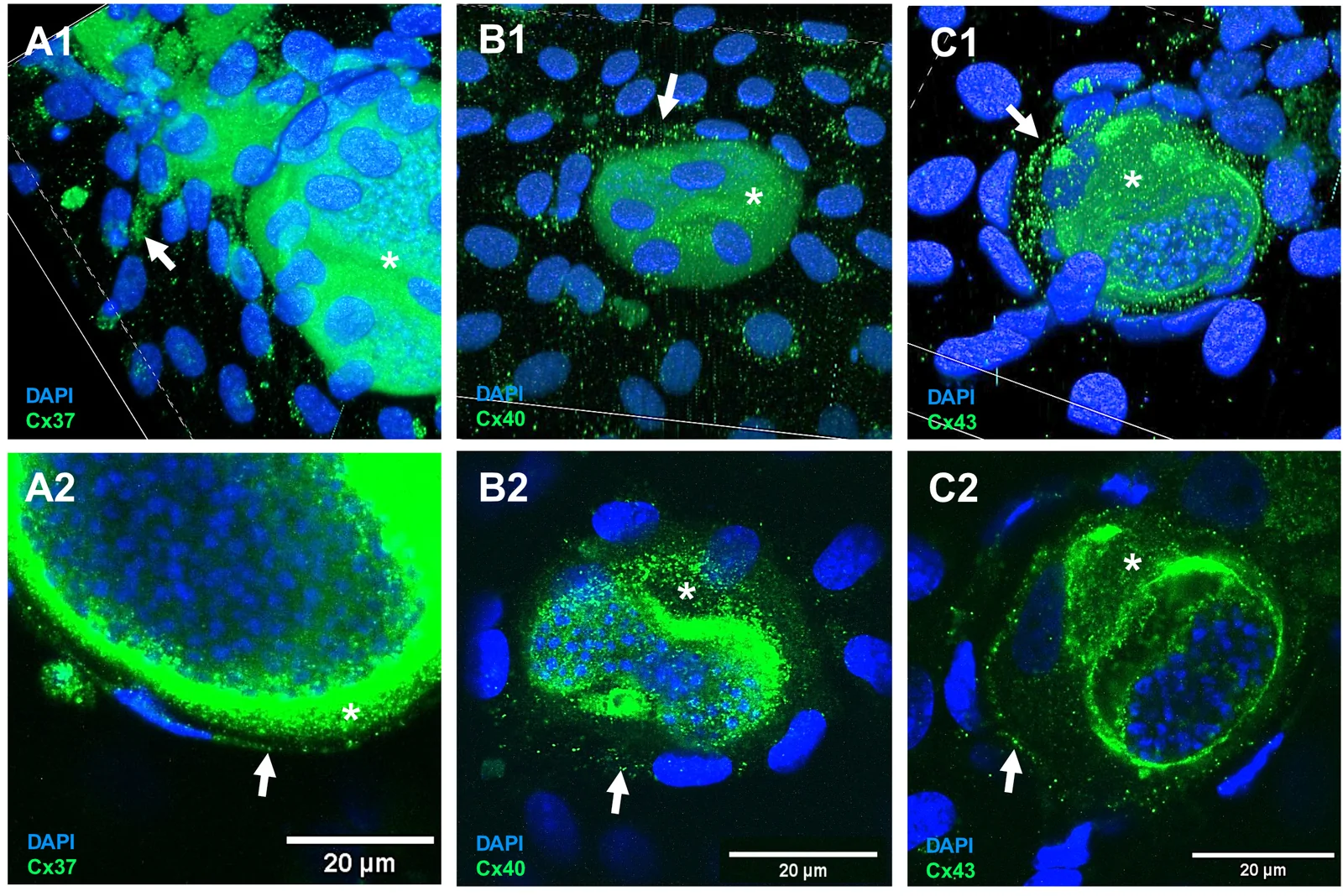
3D projections of *E. bovis*-infected BUVEC and bystander cells stained for Cx37, Cx40 and Cx43. **(A1)** Cx37 staining showing strong Cx37 signal in the MCHCs (asterisk) and encapsulating BCs (arrow). **(A2)** Z-stack slice clearly illustrating distinguishable multiple Cx37 clusters within the MCHC (asterisk) and membrane-associated plaques in adjacent BCs (arrow). **(B1)** Cx40 staining showing strong Cx40 signal and numerous multiple clusters (arrow) around the MCHC (asterisk). **(B2)** Z-stack slice of Cx40 staining showing distinguishable multifocal clusters associated with the MCHC (asterisk) and adjacent BCs (arrow). **(C1)** Cx43 staining showing multifocal and membrane-associated Cx43 clusters surrounding the MCHC (asterisk). **(C2)** Z-stack slice of Cx43 staining showing abundant Cx43 clusters in MCHC and membrane-associated Cx43 plaques between MCHC and BCs (arrow).

Besides connexin expression levels, we also intended to analyze the subcellular connexin distribution and the formation of gap junction plaques in *E. bovis*-infected cell layers. The oligomerization of connexins into hemichannels (connexons) occurs after the protein exits the ER. Connexin plaques, or gap junction plaques, are highly organized, two-dimensional dense clusters of individual gap junction channels, consisting of tens to thousands of channels connecting adjacent cells. For gap junction formation, connexons are trafficked to the cell surface, cluster into plaques, and are later removed via endocytosis. Therefore, connexin plaques do not represent permanent structures and possess a rather short half-life of 1–5 hours (Laird, 1996; Falk et al., 2014). Therefore, the likelihood of detecting connexin plaques in membranes in snapshot experiments using fixed samples could be lower than the chance to identify connexins in cytoplasmic position either on their way to the membrane or after endocytosis. In the current experimental approach, we analyzed if *E. bovis* infection and macromeront formation would induce a shift in subcellular localization, abundance and membrane-associated plaque formation of Cx37, Cx40 and Cx43 in infected or surrounding non-infected BCs. Immunostaining revealed distinct connexin patterns in non-infected and *E. bovis*-infected BUVEC layers (**Figure 3**). Overall, in non-infected control cells, few Cx37-positive globular signals were detected, which mainly showed perinuclear position, whilst Cx37-positive membrane-associated plaques were hardly found (**Figure 3A1**). The globular signals were not observed in every biological replicate, where Cx37 appeared mainly as single perinuclear cluster (**Supplementary Figure 4**). Compared to control cell layers Cx37-positive clusters were more abundant in infected cultures, particularly in BCs and non-infected cells adjacent to MCHCs (**Figure 3A2–A3**). Notably, MCHCs showed a high fluorescent signal inside. In contrast to Cx37, Cx40-positive signals were more evenly distributed across the total cytoplasm but were also rarely found membrane-associated in non-infected BUVEC (**Figure 3B1**). In *E. bovis*-infected BUVEC layers, the abundance of Cx40 signals increased but remained mainly cytoplasmic (**Figure 3B2**). Again, MCHCs revealed strong signals. In addition, a high number of Cx40-positive structures were visible in the cytoplasm of infected host cells (IHCs) outside the parasitophorous vacuole (PV) (**Figure 3B3**). In case of Cx43, positive signals were mainly found focally in perinuclear position, while membrane-associated staining was observed in some parts of the layer (**Figure 3C1**). In contrast, in infected cell layers, some cells showed more prominent Cx43 clusters and increased membrane-associated staining, whereas others retained or even lost cytosolic signals (**Figure 3C2**). Again, MCHCs displayed intense green signals (**Figure 3C3**). A combined Cx37/Cx40/Cx43 staining reflected the observations mentioned above and mainly showed perinuclear connexin signals and mild membrane staining in non-infected BUVEC (**Figure 3D1**). Meanwhile, the formation of gap junction plaques in cell membranes was more prominent in *E. bovis*-infected BUVEC layers (**Figure 3D2**), arising from merged signals of all three connexins, and being more abundant in close vicinity to MCHCs (**Figure 3D2**). Confocal 3D imaging further supported these findings and revealed accumulation of Cx37, Cx40 and Cx43 around and within MCHCs (**Figures 4A1–C1**). Although MCHCs showed partial PV-associated autofluorescence, distinguishable Cx37, Cx40 and Cx43 clusters were clearly visible outside the PV area and across different optical planes. Moreover, all three connexins showed distinct whole-cell staining patterns within MCHCs, indicating that increased connexin signal was not solely caused by PV autofluorescence (**Figures 4A2–C2**). Additionally, Z-stack analysis revealed clear membrane-associated Cx37- and Cx43 clusters at or near contact regions between MCHCs and adjacent BCs (**Figures 4A2–C2**).

**Figure 5:**
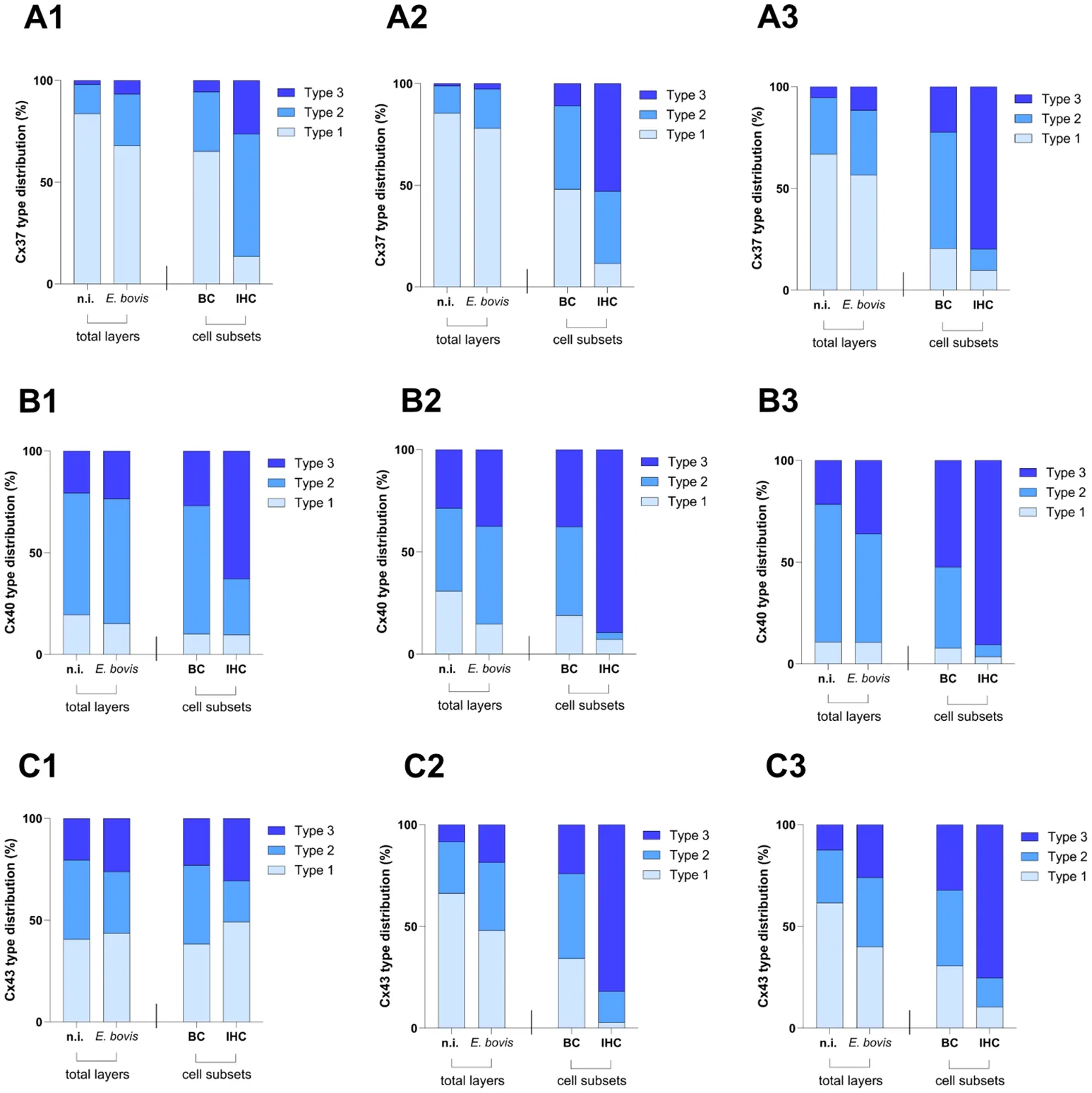
Semi-quantitative assessment of connexins (Cx) during *E. bovis* macromeront development. Cx abundance and localization was categorized as staining pattern types (type 1-3, for strategy see below) in cells of non-infected controls (n. i.) and *E. bovis-*infected layers or in single bystander cells (BC) and *E. bovis*-infected host cells (IHC) present within infected BUVEC layers. Type 1 indicates low diffuse and/or perinuclear Cx-staining with no or few (≈ 1-5) Cx clusters, type 2 indicates multiple (≈ 6-20) prominent Cx-clusters and/or low membrane-associated plaques, and type 3 refers to a high number (>20) of connexin clusters and/or prominent membrane-associated plaques. **(A1)** Cx37 at early infection, 7–8 days p. i. **(A2)** Cx37 during early meront development, 12–14 days p. i. (A3) Cx37 during late meront development, 17–18 days p. i. **(B1)** Cx40 at early infection, 7–8 days p. i. **(B2)** Cx40 during early meront development, 12–14 days p. i. (B3) Cx40 during late meront development, 17–18 days p. i. **(C1)** Cx43 at early infection, 7–8 days p. i. **(C2)** Cx43 during early meront development, 12–14 days p. i. **(C3)** Cx43 during late meront development, 17–18 days p. i. Data are shown as proportions (%) of types; numbers of individual categorized cells per graph: n ≈ 500 (total layers), n ≈ 200 (BC), n ≈ 70 (IHC) of three biological replicates.

To obtain more quantitative evidence of the staining patterns, connexin abundance and position was categorized at a semi-quantitative level in single cells (**Figures 5A1–C3**). The respective strategy is described in the Material & Method part and illustrated in the supplements (**Supplementary Figure 3**). Overall, the proportion of cells with type 2 and 3 (=higher abundance of clusters and/or increased membrane localization) increased with ongoing macromeront development for all, Cx37, Cx40 and Cx43 in both BCs and single infected cells (IHCs). Hence, *E. bovis*-infected BUVEC layers generally showed a different type of distribution for Cx37, Cx40 and Cx43 when compared to non-infected cell layers (**Figure 5**). This difference in type distribution applied to all, early infection (7–8 days p. i. with Cx43 as exception, **Figures 5A1–A3**), early MCHC development (12–13 days p. i., **Figures 5B1–B3**) and late MCHC development (17–18 days p. i., **Figures 5A3–C3**) but continuously rose with prolonged infection period. Overall, these findings indicate an infection-driven alteration of connexin abundance and/or spatial shift in BUVEC, characterized by an increased focal, multifocal and membrane-associated connexin accumulation.

### Inhibition of gap junction formation in bystander cells impairs merozoite I production

**Figure 6:**
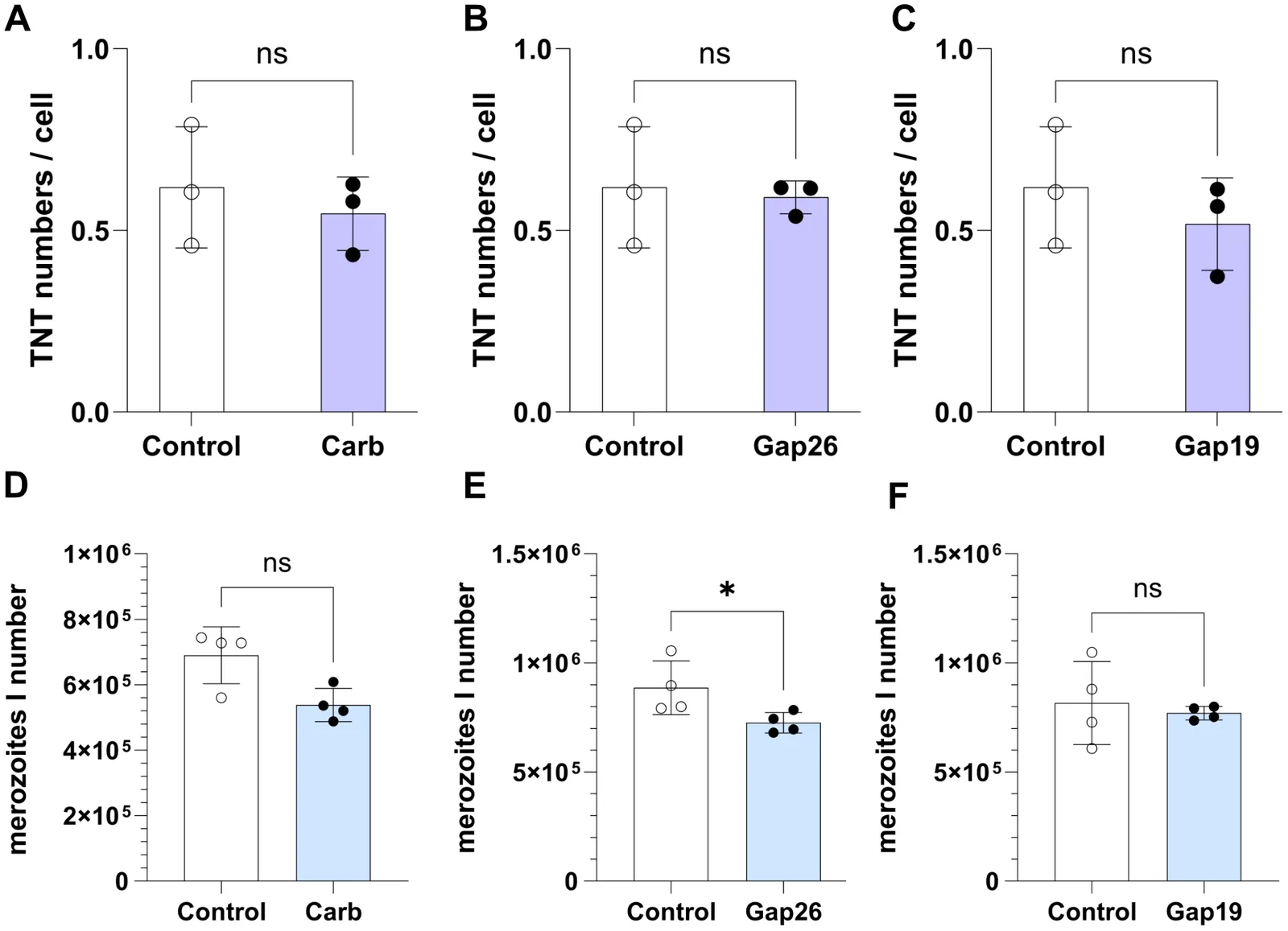
Effects of gap junction inhibition in BCs on *E. bovis* proliferation. Non-infected BUVEC were treated with carbenoxolone (Carb: **(A, D)**, Gap26 **(B, E)** or Gap19 **(C, F)** and either assessed for TNT formation 24 h post treatment **(A–C)** or co-cultured with *E. bovis* macromeront-carrying host cells (MCHCs) and analyzed for the level of merozoite I release between 15–28 days p. i. **(D–F)** Arithmetic mean and standard deviation of three to four biological replicates; ns, not significant, *p ≤ 0.05.

Given that connexins were upregulated on protein level or increasingly translocated in *E. bovis* infection, we next intended to assess the functional relevance of BC-derived gap junction formation for effective *E. bovis* macromeront development. Here, we used the meront-transfer-system to selectively pretreat non-infected BUVECs (later serving as BCs in subsequent mixed cultures with MCHCs) with inhibitors of gap junction formation. Therefore, carbenoxolone (general connexin inhibitor), Gap19 (Cx43-specific inhibitor) and Gap26 (blocks Cx37, Cx40 and Cx43) were used (Willebrords et al., 2017). As read-out for *E. bovis* development, merozoite I production was quantified. Given that connexins were already shown to be involved in TNT biogenesis [e. g. Cx43, Tishchenko et al. (2020)], we additionally studied the effects of carbenoxolone, Gap19 and Gap26 on TNT formation in non-infected BUVEC. Overall, in our hands, none of these inhibitors had significant effects on TNT formation but showed a mild tendency to downregulate TNTs (**Figures 6A–C**). Notably, when transferring MCHCs to carbenoxolone and Gap26 pre-treated BUVEC, macromeront development indeed proved impaired since merozoite I production was reduced (**Figures 6D, E**). Statistical analyses unveiled that these reactions reached a significant level only for Gap26 treatments (treated vs untreated BCs: *p ≤ 0.0282, **Figure 6E**). In contrast, but in line with protein expression profiles, BC pre-treatments with Gap19, which exclusively targets Cx43 (Willebrords et al., 2017), failed to affect parasite offspring synthesis (**Figure 6F**). Overall, these data indicated a key role of Cx37 and Cx40, but not of Cx43, for effective parasite proliferation.

### Mitochondria donation from non-infected bystander cells to *Eimeria bovis*-infected host cells is a common event

**Figure 7:**
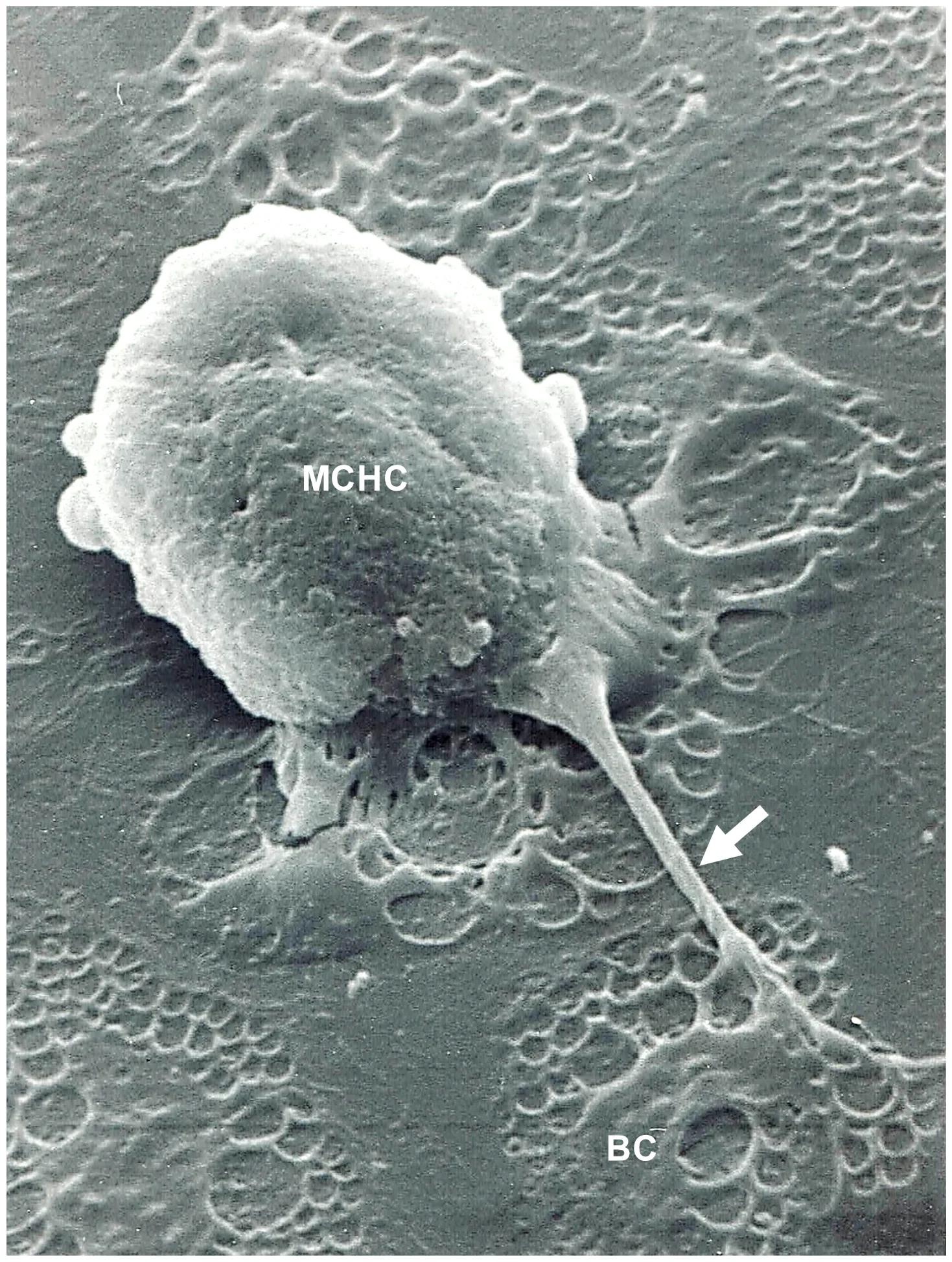
SEM illustration of a TNT (arrow) being formed between an *E. bovis* meront-carrying host cell (MCHC, 12 days p. i.) and a non-infected bystander cell (BC).

**Figure 8:**
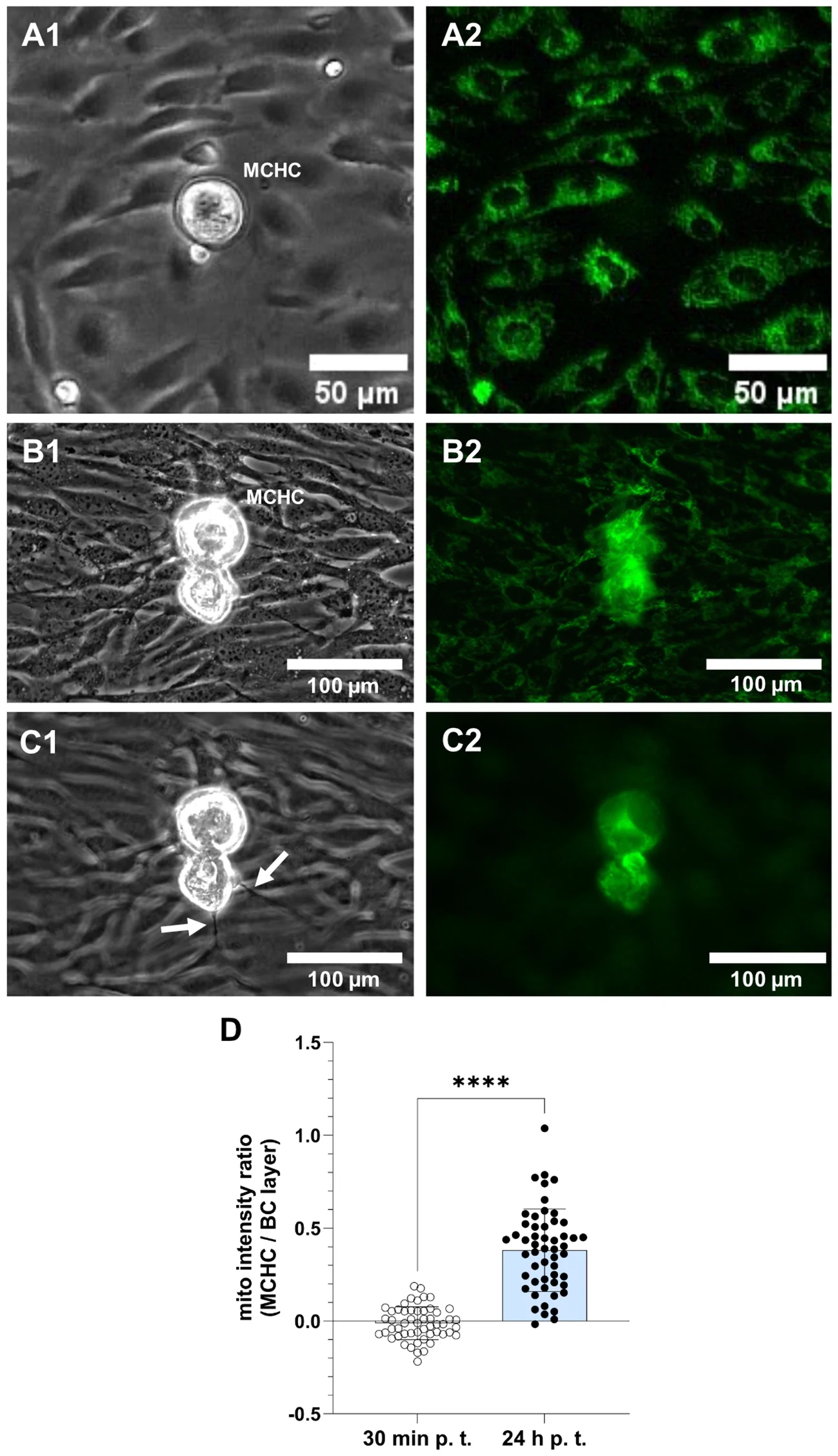
Transfer of mitochondria from bystander cells (BCs) to *E. bovis*-infected host cells (MCHCs). After isolation, unstained MCHCs were transferred to MitoView™Green-stained non-infected BUVEC, serving as BCs. Phase contrast **(A1)** and fluorescence **(A2)** illustration of an MCHC 30 min post transfer (p. t.). Note that the MCHC is lacking fluorescence, indicating that the fluorescent dye showed no leakage. **(B, C)** illustration of an MCHC integrated into the BC layer at 24 h (p) t., focused at the z-plane of BCs **(B1, B2)** or of the MCHC **(C1, C2)**, showing two TNTs being formed between the MCHC and BCs (white arrows). The green fluorescence of the MCHC **(C2)** proves the uptake of stained mitochondria from BCs. **(D)** Quantification of MCHCs that obtained mitochondria from the MitoView™Green-stained recipient BC layer. Arithmetic mean and standard deviation of 53 MCHCs of three biological replicates; ****p ≤ 0.0001.

To validate and expand previous observations on TNT-mediated mitochondrial transfer (Fischer et al., 2025), we here first confirmed the TNT formation between MCHCs and BUVECs on SEM level (**Figure 7**). Hence, a thin, elongated membrane protrusion extending from an MCHC towards an adjacent BC was shown at 12 days p. i. (**Figure 7**). Given that this structure hovered over the substrate (i. e. BUVEC layer) and established a direct cell-to-cell contact spanning several micrometers, it was structurally consistent with a TNT. Moreover, we here assessed mitochondrial donation from BCs to *E. bovis*-infected host cells (MCHCs) on a quantitative level. Therefore, exclusively mitochondria of non-infected BUVEC serving as BCs were stained by MitoView™Green before MCHC supplementation. To control for potential dye leakage, MCHCs were monitored for fluorescence early after transfer (30 min), i. e. at a time point when MCHCs had not yet integrated into the BUVEC layer and could therefore not establish TNT formations but were exposed to supernatants potentially containing leakage-borne mitochondrial dye. Of note, MitoView™Green stains mitochondria within seconds to minutes and at very low concentrations (< 20 nM; biotium.com/product/mitoview-dyes). As illustrated in **Figures 8A1, A2**, MCHCs showed no fluorescence at 30 min post transfer, thereby confirming the validity of the current staining method by a lack of dye carryover. We then tested for mitochondria uptake by MCHCs at 24 h post MCHC supplementation, i. e. when MCHCs had successfully integrated into the recipient BC layer, thereby being able to communicate with adjacent cells. At this stage, MitoView™Green-labeled mitochondria, which must have originated from stained non-infected BUVECs, were clearly detectable in MCHCs (**Figures 8B2, C2**). The abundance of MitoView™Green-labeled mitochondria in MCHCs increased significantly between 30 min and 24 h post transfer (*p ≤ 0.0001, **Figure 8D**). On quantitative level, MCHC uptake of mitochondria from adjacent cells signified a common event, since it occurred in 89% of MCHCs (*n* = 47/53 with three biological replicates, **Figure 8D**). Moreover, TNTs connecting MitoView™Green-positive MCHCs with BCs were simultaneously detected (**Figure 8C1**, arrows), consistent with mitochondrial transfer via these structures.

### Microtubule inhibition and cellular stress modulation in bystander cells alter merozoite I production

**Figure 9:**
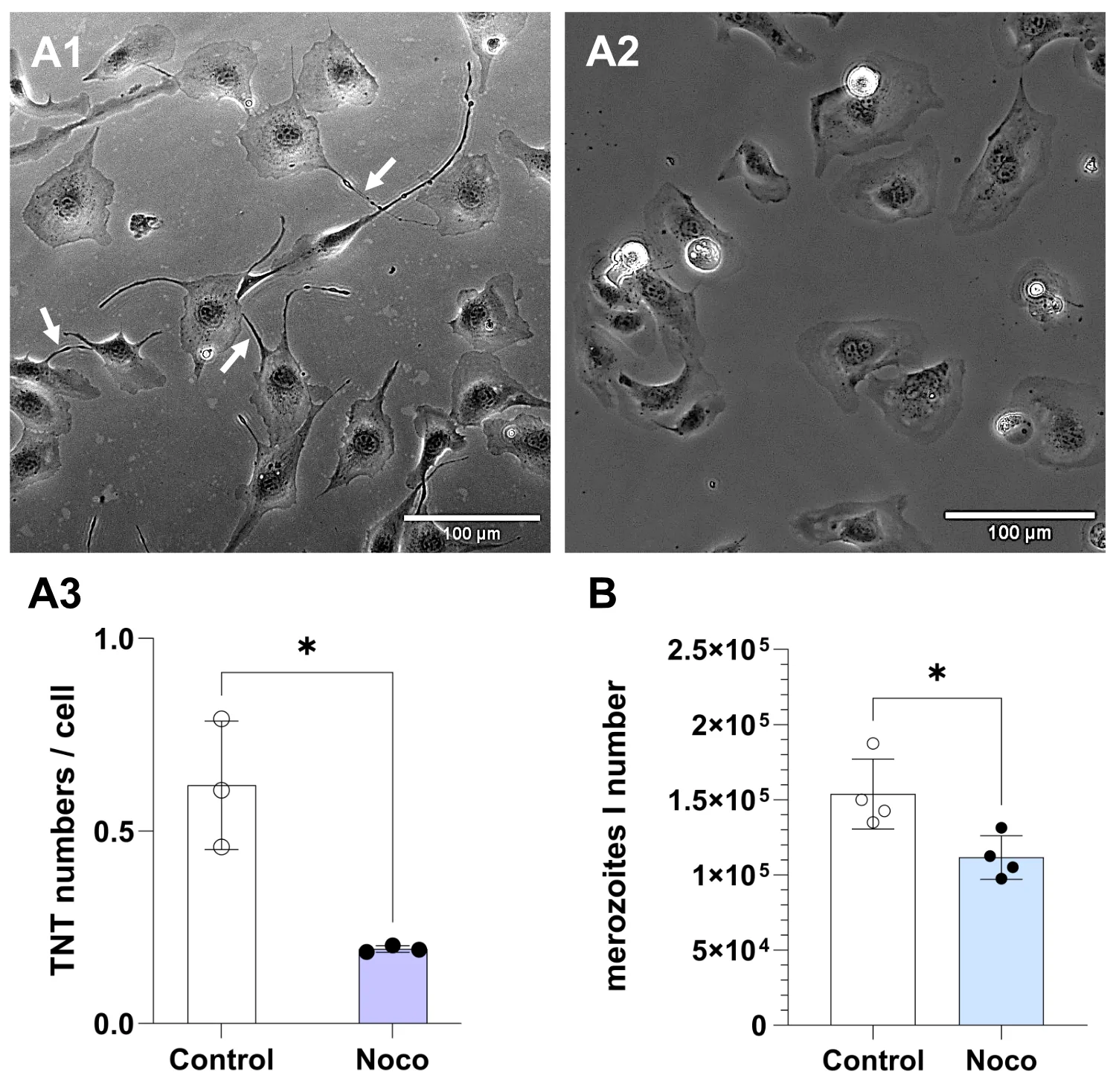
Effects of BC nocodazole treatments on TNT formation and on *E. bovis* proliferation. **(A1, A2)** exemplary illustration of TNT formation (arrows) in non-treated control cells **(A1)** and in nocodazole-treated BUVEC **(A2)**. **(A3)** quantification of TNT formation in nocodazole-treated BUVEC. **(B)** Effects of nocodazole BC pretreatments on *E. bovis* merozoite I production after MCHC transfer. Arithmetic mean and standard deviation of three to four biological replicates; *p ≤ 0.05.

**Figure 10:**
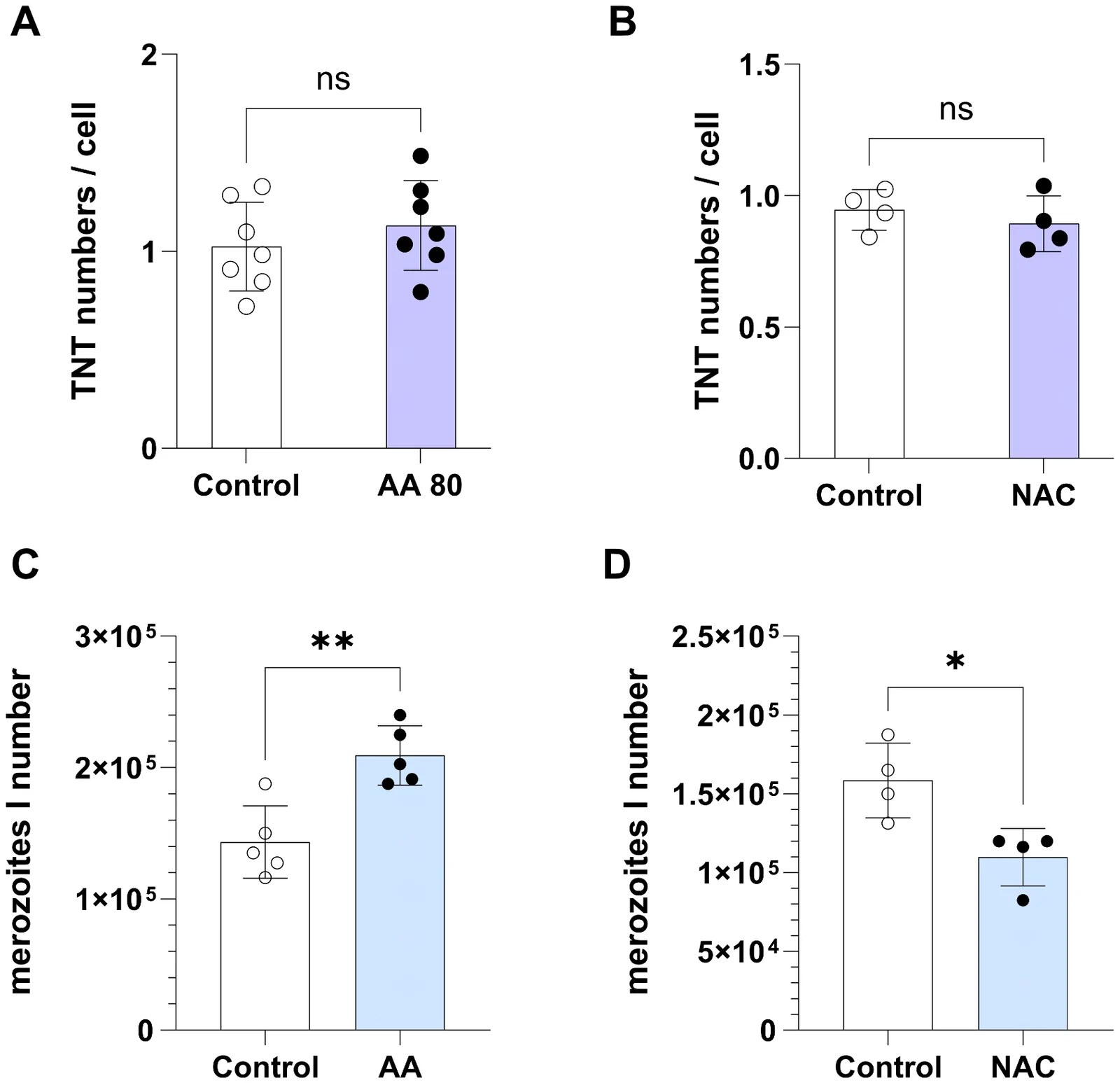
Effects of arachidonic acid (AA) and _N_-acetylcysteine (NAC) treatments of BCs on TNT formation and on *E. bovis* proliferation. Quantification of TNT formation in **(A)** arachidonic acid- and **(B)**_N_-acetylcysteine-treated BUVEC. Quantification of merozoites I in mixed cultures of arachidonic acid- **(C)** and _N_-acetylcysteine-pretreated **(D)** BUVEC (serving as BCs) and macromeront-carrying host cells (MCHCs). arithmetic mean and standard deviation of four to seven biological replicates; *p ≤ 0.05, **p ≤ 0.005.

As a well-documented characteristic, TNTs are rich in F-actin cytoskeletal filaments but may also contain microtubules (Austefjord et al., 2014). Given that blockage of actin dynamics by cytochalasin B treatments reduced TNT formation and impaired *E. bovis* macromeront development in BUVEC (Fischer et al., 2025) and based on above mentioned findings, we here studied if microtubules as rarer components of TNTs were also crucial for effective macromeront formation. Therefore, the classical microtubule inhibitor nocodazole was used at a low concentration of 50 nM for non-infected BUVEC treatments before meront transfer. In this setting, TNT formation was not entirely blocked but significantly reduced by 69% in BUVEC (treated vs. non-treated BUVEC: *p = 0.0476, **Figures 9A1-A3**). Previous findings had shown that the host cellular microtubule cytoskeleton was significantly affected by *E. bovis* infection since microtubules were re-arranged and increased in abundance with ongoing macromeront formation (Hermosilla et al., 2008). To avoid any interference of nocodazole treatments with *E. bovis* development and to test the role of functional microtubules potentially forming part of TNTs in BCs, we here again profited from the meront-transfer-system to exclusively treat recipient non-infected BUVEC (= BCs) with nocodazole before transferring MCHCs. As expected, subsequent *E. bovis* macromeront development was indeed impaired in this experimental approach since merozoite I production was significantly diminished (nocodazole-treated vs. non-treated BCs: *p ≤ 0.0286, **Figure 9B**). Unfortunately, specific stimulants and inhibitors of TNT formation are currently lacking. Nevertheless, we here additionally tested other compounds that were described to affect TNT formation. Hence, arachidonic acid (AA) was reported to enhance TNT formation, whilst _N_-acetylcysteine (NAC) induced the opposite effect in other experimental settings (Astanina et al., 2015; Lin et al., 2024). When using arachidonic acid (80 µM) for non-infected BUVEC treatments, TNTs were only mildly upregulated by 13% (treated vs untreated cells: **Figure 10A**), which is partially in accordance with findings in human microvascular endothelial cells-1, but did not reach the significance level in this experimental set up (Astanina et al., 2015). In contrast, treatments of BUVEC with NAC (1 µM) failed to significantly reduce TNT formation even though a tendency of reduction was observed (7%, **Figure 10B**). Nevertheless, when transferring non-treated MCHCs to arachidonic acid- (80 µM) and NAC-treated (1 µM) BUVEC, both BC treatments significantly affected parasite proliferation. Arachidonic acid upregulated merozoite I production (treated BCs vs. untreated controls: *p ≤ 0.003, **Figure 10C**), whilst NAC led to diminished offspring synthesis (treated BCs vs. untreated controls: *p ≤ 0.0286, **Figure 10D**).

### Inhibition of selected metabolic pathways in bystander cells diminishes merozoite I production

**Figure 11:**
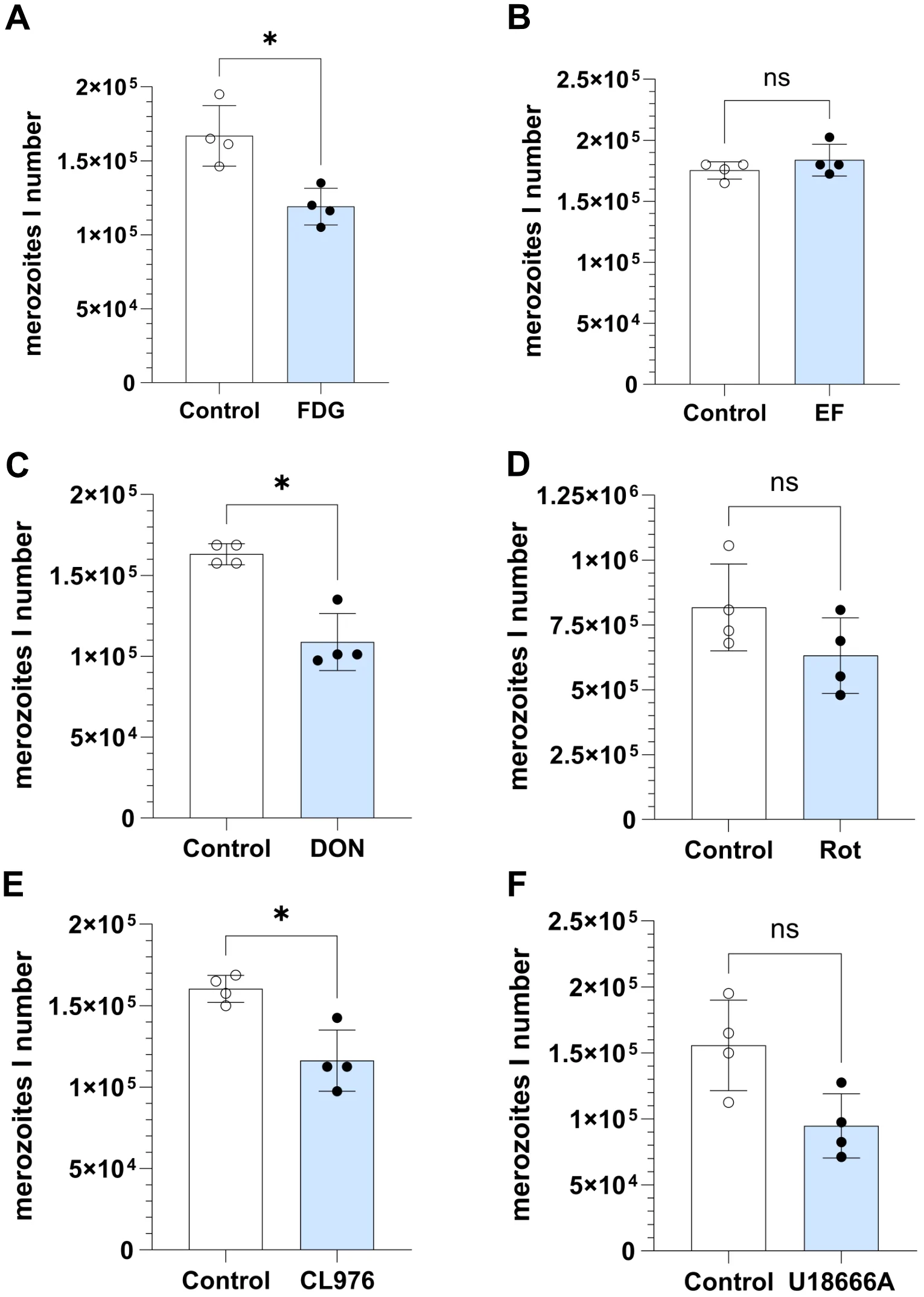
Effects of selected metabolic pathway inhibition in BCs on *E. bovis* proliferation. Effects of **(A)** fluordeoxyglucose(^18^F) (FDG), **(B)** ethylfluoracetate (EF), **(C)** 6-Diazo-5-oxo-L-norleucine (DON), **(D)** rotenone (Rot), **(E)** CL976 and **(F)** U18666A pretreatments of recipient BUVEC (= BCs) on merozoite I production after MCHC transfer. Arithmetic mean and standard deviation of four biological replicates; *p ≤ 0.05.

Cellular actions like TNT biogenesis or mitochondrial donation require energy. To furthermore identify key metabolic requirements of BC-mediated MCHC support, we treated non-infected BCs with inhibitors of selected metabolic pathways prior to MCHC supplementation using the meront-transfer-system (**Figures 11A–F**). Here, the pharmacological blockage of glycolysis and glutaminolysis in BCs by ^18^F-fluordeoxyglucose (FDG, 100 µM, **Figure 11A**) and 6-diazo-5-oxo-L-norleucine (DON, 2 µM, **Figure 11C**) treatments, respectively, resulted in a significant reduction of merozoite I production (FDG and DON vs control: both *p ≤ 0.0286). In contrast, the inhibition of the TCA cycle by ethylfluoracetate (EF, 1 mM, **Figure 11B**) and of the mitochondrial electron transport chain (complex I) by rotenone (1 µM, **Figure 11D**) failed to significantly affect merozoite I production, although rotenone showed a tendency to lower merozoite I production. Given that *E. bovis* macromeront formation is well-documented to depend on the host cellular cholesterol metabolism and trafficking (Hamid et al., 2014, Hamid et al., 2015; Silva et al., 2022), we here also tested if the inhibition of cholesterol acyltransferase activity by CL976 (5 µM, **Figure 11E**) and of intracellular cholesterol transport by U18666A *(*2 µM, **Figure 11F**) in BCs affected subsequent macromeront formation. Here, both pre-treatments of BCs led to a reduction of merozoite I production, however, merely CL976 treatments reached statistical significance (CL976 vs. control: *p* ≤ 0.0286). Overall, these data show that BC-mediated MCHC support requires intact glycolytic- and glutaminolytic activities in addition to functional cholesterol metabolism and trafficking in BCs.

## Discussion

Recent findings on *E. bovis* macromeront formation evidenced that non-infected BCs accumulate around *E. bovis*-infected primary host endothelial cell and donate mitochondria via TNTs *in vitro* (Fischer et al., 2025). We here expanded the current knowledge on intercellular communication between infected host cells and BC by showing that gap junction formation is upregulated in *E. bovis*-infected cell layers, potentially enabling BCs to donate small molecules to MCHCs. Accordingly, we reported that effective *E. bovis* macromeront formation with merozoite I production requires intact glycolytic- and glutaminolytic activities in addition to a functional cholesterol metabolism and transport in BCs. Moreover, we demonstrated on a quantitative level that TNT-based mitochondria donation from BCs to MCHCs is a common process, most likely serving to restore mitochondrial dysfunction in MCHCs. By the current findings, we emphasize the key role of a structurally and metabolically intact cellular environment for successful *E. bovis* macromeront formation, confirming the novel concept of multicellular environmental molecule and organelle donation for intracellular parasite proliferation sustainment as recently reported (Fischer et al., 2025).

One key mechanism of intercellular communication is gap junction-based cell coupling and molecule transfer. The connexins Cx37, Cx40, Cx43 are the major gap junction proteins expressed in endothelial cells (Van Rijen et al., 1997). Gap junction channels allow for the transfer of hydrophilic molecules <1000 Da, hence, small molecules can be transferred freely between cells depending on the cytoplasmic concentration gradient (Kumar and Gilula, 1996). In principle, different combinations of these connexins promote the formation of homo- or heteromeric connexons and homo- or heterotypic gap junctions leading to a variety of channel types with different functional properties (Veenstra, 1996; Ebong et al., 2006). In the current work, kinetic profiling of connexin expression in *E. bovis*-infected primary bovine endothelial cells revealed a consistent upregulation of Cx37- and Cx40 protein expression during macromeront formation. Surprisingly, despite its well-documented involvement in stress, inflammation and remodeling responses in primary human umbilical vein endothelial cells (HUVEC), Cx43 expression was not affected significantly in protein abundance by *E. bovis* infection, indicating a more selective engagement of Cx40/Cx37-mediated interactions (Scheckenbach et al., 2011; Chen et al., 2015; Okamoto et al., 2021). In line, coordinated changes in Cx40 and Cx37 expression were reported for HUVEC showing that Cx40 is co-expressed with Cx37 in larger vessels and that Cx40 promotes and stabilizes Cx37 expression (Looker et al., 2023). Moreover, in contrast to a modest relevance of Cx43, Cx40 and Cx37 seemed of major importance in endothelial cell coupling (Ebong et al., 2006) and were involved in limiting endothelial cell proliferation (Looker et al., 2023). Accordingly, *E. bovis*-infected BUVEC are impaired in cell cycling and do not divide after infection (Velásquez et al., 2021a).

Given that total infected cell layers containing a low proportion of infected cells and a high proportion of non-infected cells (the infection rate varies with infection duration and accounts for 2 - 7% at meront level) were processed in the current study for western blotting, we cannot deduce from these experiments if upregulation of connexin expression was attributed to non-infected cells or infected cells or both within the cell layer. However, the finding that gap junction inhibition in BCs resulted in impaired parasite proliferation in subsequent mixed cultures supports a role for gap-junction-mediated coupling between infected and non-infected cells. In line with connexin expression, only pre-treatments of BCs with carbenoxolone (general gap junction blocker) and Gap26 (targets Cx37, Cx40 and Cx43), but not with Gap19 (blocks Cx43), significantly diminished merozoite I production in mixed MCHC/BC cultures (Willebrords et al., 2017). Additionally, connexin immunostaining of infected cell layers complemented the western blot data by revealing connexin-dependent changes in protein abundance and infection-driven connexin translocation. In line with increased protein levels of Cx37 and Cx40 detected by western blotting, an increased cytosolic presence and clustering of these connexins was detected in *E. bovis*-infected layers. The perinuclear/Golgi-associated, vesicular and membrane-associated connexin staining observed here is consistent with the known trafficking (ER/Golgi), membrane insertion, internalization, recycling and degradation of connexins, including connexosome/annular gap junction formation (Aasen et al., 2019; Bell et al., 2019; Norris and Terasaki, 2021). In contrast to Cx37 and Cx40, western blot-based data showed no infection-driven upregulation of Cx43 expression. However, immunostaining of Cx43 revealed a translocation of Cx43 into cell membranes in addition to an upregulated Cx43-based gap junction plaque formation, suggesting a potential role of Cx43 in functional gap junction-mediated small molecule transfer in infected cells. A similar Cx43 distribution pattern, including stress-induced trafficking to cell membranes, has been reported in neonatal myofibroblasts (Johansen et al., 2011). In line with current connexin findings, previous studies on endothelial cells also reported Cx37-, Cx40- and Cx43 staining at cell-cell interfaces, but additionally showed perinuclear or intracellular signals, supporting the current hypothesis that connexin staining patterns reflected connexin trafficking and recruitment to cell membranes (Ebong et al., 2006; Meens et al., 2015; Mannell et al., 2021). Thus, we conclude that connexin expression induction and spatial remodeling - and thereby endothelial cell coupling by gap junction formation - was boosted in *E. bovis*-infected cell layers to enable a BC-driven transfer of small molecules such as metabolites, second messengers, ATP or ions (Evans and Martin, 2002) to MCHCs. So far, the nature of these molecules is not defined. Notably, in another context, hematopoietic stem cells (HSCs) were recently shown to donate several amino acids, glycolytic- and tricarboxylic acid cycle metabolites to damaged vascular endothelial cells as energy sources (Ogawa et al., 2022). For *E. bovis* macromeront development we recently stated both enhanced energy demands and boosted glycolytic activities and demonstrated that MCHCs experienced a critical metabolic state leading to premature senescence (Velásquez et al., 2021b). Besides other metabolites, the transfer of ATP as a major source of energy is a well-documented feature of gap junction opening (Goldberg et al., 2002; Harris, 2007), which could also help to restore parasite-driven bioenergetic impairment in MCHCs (Velásquez et al., 2021a). Moreover, low-molecular weight water-soluble molecules such as glucose can freely pass through gap junctions when a concentration gradient is present and may therefore be obtained from donor cells as a prerequisite for enhanced glycolytic activities, which were already documented for MCHCs (Velásquez et al., 2021b). Notably, a gap junction-based transfer of a glucose homologue to primary HUVEC was recently reported (Kikuchi-Taura et al., 2020). In agreement with enhanced glycolytic requirements during *E. bovis* macromeront development (Velásquez et al., 2021b), the selective blockage of glycolysis in BCs via FDG treatments significantly reduced merozoite I synthesis in MCHC-BC-cocultures, thereby indicating that intact glycolytic activities in BCs were key to BC-mediated MCHC support. Besides glycolysis, the relevance of other key metabolic pathways (TCA cycle, glutaminolysis, mitochondrial respiration, cholesterol esterification and transport) was assessed in BC inhibition experiments to identify further metabolic requirements of BC-MCHC-interactions. Here, statistical analyses identified a significant role of intact glutaminolysis and cholesterol esterification activities in BCs, since treatments with DON and CL976, respectively, resulted in a marked reduction of merozoite I production. Referring to glutaminolysis, the effects seem consistent with the well-known role of glutaminolysis (besides glycolysis) for energy generation in bovine endothelial cells (Leighton et al., 1987). Notably, glutamine represents the most abundant amino acid in blood plasma, providing endothelial cells with substrates for energy production. However, glutamine itself, as well as the glutaminolytic intermediates glutamate and aspartate, also function as precursors for nucleic acid biosynthesis. Furthermore, acetyl-CoA generated from glutaminolysis-derived pyruvate can also be utilized for *de novo* fatty acid synthesis (Hosios et al., 2016; Yoo et al., 2020). Thus, several metabolites of the glutamine metabolism seem beneficial for macromeront development. Referring to cholesterol metabolism, it is well-documented that coccidian parasites are defective in *de novo* cholesterol synthesis (Bansal et al., 2005; Labaied et al., 2011; Coppens, 2013; Ehrenman et al., 2013). Accordingly, *E. bovis* was shown to foster both host cellular *de novo* cholesterol synthesis and LDL-based lipid uptake from the environment during merogony I (Hamid et al., 2014; Taubert et al., 2018; Silva et al., 2022). Referring to current results, cholesterol esterification may be pivotal to fulfil enhanced demands in BC driven either by TNT-based membrane synthesis or by vesicle-based trafficking processes. In line, the blockage of intracellular cholesterol transport in BCs by U18666A also reduced merozoite I production but failed to reach a significant level. Notably, the same inhibitor significantly blocked macromeront development when directly applied to *E. bovis*-infected endothelial cells (Silva et al., 2022). Moreover, images by Silva et al. (2022) suggest enhanced cholesterol staining also in BCs and not exclusively in MCHCs. Therefore, current findings underscore the importance of a functional cholesterol metabolism not only for host cells but also for the cellular environment of MCHCs.

Connexin-based gap junctions and TNT formation constitute different modes of intercellular communication but also seem to be linked since connexins have also been implicated in TNT biogenesis. Notably, Cx43 localizes to TNTs and stabilizes these structures in breast cancer cells (Tishchenko et al., 2020). Moreover, the gap junction blocker meclofenamate inhibits TNT-mediated voltage-sensitive electrical coupling in rat kidney cells (Wang et al., 2010). Since it seemed conceivable that endothelial connexins not only contributed to direct metabolite transfer but also to the integrity of TNT connections, we studied if gap junction inhibition also affected TNT formation in bovine endothelial cells. However, neither Gap19, Gap26 nor carbenoxolone treatments significantly affected TNT formation, which was recently proven to be upregulated during *E. bovis* macromeront formation (Fischer et al., 2025). Moreover, the relevance of TNTs for effective macromeront formation was demonstrated by cytochalasin B treatments of BCs, which reduced both TNT formation and merozoite I generation (Fischer et al., 2025). To validate the role of further TNT components, we here blocked microtubules in BCs by nocodazole treatments. Microtubule dynamics are key to intracellular trafficking and membrane organization, and microtubules form part of distinct TNT types (Austefjord et al., 2014; Zhao et al., 2024). Nanomolar nocodazole treatments, which did not affect cell viability, were surprisingly potent in reducing TNT formation (69%) in non-infected BUVEC. Moreover, BC treatments led to a significant diminishment of merozoite I production in mixed BC/MCHC cultures suggesting TNT formation as relevant for BC-MCHC-interactions. By also applying a stimulant of TNT formation with arachidonic acid, a regulator of membrane dynamics and signaling, which was previously shown to promote TNT formation (Astanina et al., 2015), we observed improved intracellular parasite development after BC treatment, although TNT formation itself was only mildly increased. To analyze TNT-based mechanism in more detail, the donation of mitochondria from BC to MCHC was quantitatively assessed. In line with the recent proof of concept (Fischer et al., 2025), the TNT-based mitochondria transfer was identified as a common mechanism to potentially support metabolically stressed *E. bovis*-infected host cells since a high proportion of MCHCs (89%) obtained mitochondria from BCs. It is tempting to hypothesize that functional BC-derived mitochondria will integrate into the mitochondrial network of stressed MCHCs experiencing mitochondrial dysfunction (Velásquez et al., 2021b) thereby helping to restore cellular function as reported in other cellular processes (Yasuda et al., 2010; Liu et al., 2014; Walters and Cox, 2021).

In summary, current findings expand the concept of multicellular environmental support of *E. bovis*-infected host cells within a cell layer by confirming gap junction and TNT formation as complementary mechanisms of intercellular molecule and organelle donation. This mechanism may represent a general principle of macromeront-forming pathogenic ruminant coccidia (e. g. *Eimeria zuernii, Eimeria ninakohlyakimovae, Eimeria arloingi, Eimeria ovinoidalis*) and suggests that these intracellular parasites manipulate not only their host cells but also their cellular microenvironment.

## Data Availability

The original contributions presented in the study are included in the article/**Supplementary Material**. Further inquiries can be directed to the corresponding author.

## References

[B1] Aasen T. Leithe E. Graham S. V. Kameritsch P. Mayán M. D. Mesnil M. . (2019). Connexins in cancer: bridging the gap to the clinic. Oncogene 38, 4429–4451. doi: 10.1038/s41388-019-0741-6 30814684 PMC6555763

[B2] Astanina K. Koch M. Jüngst C. Zumbusch A. Kiemer A. K. (2015). Lipid droplets as a novel cargo of tunnelling nanotubes in endothelial cells. Sci. Rep. 5, 11453. doi: 10.1038/srep11453 26095213 PMC4476149

[B3] Austefjord M. W. Gerdes H.-H. Wang X. (2014). Tunneling nanotubes: diversity in morphology and structure. Commun. Integr. Biol. 7, e27934. doi: 10.4161/cib.27934 24778759 PMC3995728

[B4] Bansal D. Bhatti H. S. Sehgal R. (2005). Role of cholesterol in parasitic infections. Lipids Health Dis. 4, 10. doi: 10.1186/1476-511X-4-10 15882457 PMC1142336

[B5] Bell C. L. Shakespeare T. I. Smith A. R. Murray S. A. (2019). Visualization of annular gap junction vesicle processing: the interplay between annular gap junctions and mitochondria. Int. J. Mol. Sci. 20, 44. doi: 10.3390/ijms20010044 30583492 PMC6337258

[B6] Campos de Carvalho A. C. Roy C. Hertzberg E. L. Tanowitz H. B. Kessler J. A. Weiss L. M. . (1998). Gap junction disappearance in astrocytes and leptomeningeal cells as a consequence of protozoan infection. Brain Res. 790, 304–314. doi: 10.1016/s0006-8993(97)01523-0 9593958

[B7] Cervantes D. C. Zurzolo C. (2021). Peering into tunneling nanotubes—the path forward. EMBO J. 40, e105789. doi: 10.15252/embj.2020105789 33646572 PMC8047439

[B8] Chen C.-H. Mayo J. N. Gourdie R. G. Johnstone S. R. Isakson B. E. Bearden S. E. (2015). The connexin 43/ZO-1 complex regulates cerebral endothelial F-actin architecture and migration. Am. J. Physiol. Cell Physiol. 309, C600–C607. doi: 10.1152/ajpcell.00155.2015 26289751 PMC4628939

[B9] Coppens I. (2013). Targeting lipid biosynthesis and salvage in apicomplexan parasites for improved chemotherapies. Nat. Rev. Microbiol. 11, 823–835. doi: 10.1038/nrmicro3139 24162026

[B10] Daugschies A. Najdrowski M. (2005). Eimeriosis in cattle: current understanding. J. Vet. Med. Ser. B 52, 417–427. doi: 10.1111/j.1439-0450.2005.00894.x 16364016

[B11] Dubey J. P. (2018). A review of coccidiosis in water buffaloes (Bubalus bubalis). Veterinary Parasitol. 256, 50–57. doi: 10.1016/j.vetpar.2018.04.005 29887030

[B12] Ebong E. E. Kim S. DePaola N. (2006). Flow regulates intercellular communication in HAEC by assembling functional Cx40 and Cx37 gap junctional channels. Am. J. Physiol. Heart Circ. Physiol. 290, H2015–H2023. doi: 10.1152/ajpheart.00204.2005 16361362

[B13] Ehrenman K. Wanyiri J. W. Bhat N. Ward H. D. Coppens I. (2013). Cryptosporidium parvum scavenges LDL-derived cholesterol and micellar cholesterol internalized into enterocytes. Cell. Microbiol. 15, 1182–1197. doi: 10.1111/cmi.12107 23311949 PMC3644335

[B14] Evans W. H. Martin P. E. M. (2002). Gap junctions: structure and function (review). Mol. Membr. Biol. 19, 121–136. doi: 10.1080/09687680210139839 12126230

[B15] Falk M. M. Kells R. M. Berthoud V. M. (2014). Degradation of connexins and gap junctions. FEBS Lett. 588, 1221–1229. doi: 10.1016/j.febslet.2014.01.031 24486527 PMC3989500

[B16] Fiege N. Klatte D. Kollmann D. Zahner H. Bürger H. J. (1992). Eimeria bovis in cattle: colostral transfer of antibodies and immune response to experimental infections. Parasitol. Res. 78, 32–38. doi: 10.1007/BF00936178 1584744

[B17] Fischer J. Sous L. Velásquez Z. D. Hermosilla C. Taubert A. (2025). Intracellular Eimeria bovis macromeront formation induces bystander cell accumulation and TNT formation. Front. Cell. Infect. Microbiol. 15. doi: 10.3389/fcimb.2025.1665269 41112577 PMC12531633

[B18] Goldberg G. S. Moreno A. P. Lampe P. D. (2002). Gap junctions between cells expressing connexin 43 or 32 show inverse permselectivity to adenosine and ATP*. J. Biol. Chem. 277, 36725–36730. doi: 10.1074/jbc.M109797200 12119284

[B19] Guo R. Si R. Scott B. T. Makino A. (2017). Mitochondrial connexin40 regulates mitochondrial calcium uptake in coronary endothelial cells. Am. J. Physiol. Cell Physiol. 312, C398–C406. doi: 10.1152/ajpcell.00283.2016 28122731 PMC5407023

[B20] Hamid P. H. Hirzmann J. Hermosilla C. Taubert A. (2014). Differential inhibition of host cell cholesterol de novo biosynthesis and processing abrogates Eimeria bovis intracellular development. Parasitol. Res. 113, 4165–4176. doi: 10.1007/s00436-014-4092-5 25199551

[B21] Hamid P. H. Hirzmann J. Kerner K. Gimpl G. Lochnit G. Hermosilla C. R. . (2015). Eimeria bovis infection modulates endothelial host cell cholesterol metabolism for successful replication. Vet. Res. 46, 100. doi: 10.1186/s13567-015-0230-z 26395984 PMC4579583

[B22] Hammond D. M. Bowman G. W. Davis L. R. Simms B. T. (1946). The endogenous phase of the life cycle of Eimeria bovis. J. Parasitol. 32, 409–427. doi: 10.2307/3272876 20996742

[B23] Hammond D. M. Ernst J. V. Miner M. L. (1966). The development of first generation schizonts of Eimeria bovis*. J. Protozool. 13, 559–564. doi: 10.1111/j.1550-7408.1966.tb01964.x 40046247

[B24] Harris A. L. (2007). Connexin channel permeability to cytoplasmic molecules. Prog. Biophys. Mol. Biol. 94, 120–143. doi: 10.1016/j.pbiomolbio.2007.03.011 17470375 PMC1995164

[B25] Hermosilla C. Ruiz A. Taubert A. (2012). Eimeria bovis: an update on parasite-host cell interactions. Int. J. Med. Microbiol. 302, 210–215. doi: 10.1016/j.ijmm.2012.07.002 22925990

[B26] Hermosilla C. Schröpfer E. Stowasser M. Eckstein-Ludwig U. Behrendt J. H. Zahner H. (2008). Cytoskeletal changes in Eimeria bovis-infected host endothelial cells during first merogony. Vet. Res. Commun. 32, 521–531. doi: 10.1007/s11259-008-9054-x 18668335

[B27] Hosios A. M. Hecht V. C. Danai L. V. Johnson M. O. Rathmell J. C. Steinhauser M. L. . (2016). Amino acids rather than glucose account for the majority of cell mass in proliferating mammalian cells. Dev. Cell 36, 540–549. doi: 10.1016/j.devcel.2016.02.012 26954548 PMC4766004

[B28] Jahnke R. Matthiesen S. Zaeck L. M. Finke S. Knittler M. R. (2022). Chlamydia trachomatis cell-to-cell spread through tunneling nanotubes. Microbiol. Spectr. 10, e02817-22. doi: 10.1128/spectrum.02817-22 36219107 PMC9769577

[B29] Jansens R. J. J. Tishchenko A. Favoreel H. W. (2020). Bridging the gap: virus long-distance spread via tunneling nanotubes. J. Virol. 94, e02120-19. doi: 10.1128/JVI.02120-19 32024778 PMC7108841

[B30] Johansen D. Cruciani V. Sundset R. Ytrehus K. Mikalsen S.-O. (2011). Ischemia induces closure of gap junctional channels and opening of hemichannels in heart-derived cells and tissue. Cell. Physiol. Biochem. 28, 103–114. doi: 10.1159/000331719 21865853

[B31] Kikuchi-Taura A. Okinaka Y. Takeuchi Y. Ogawa Y. Maeda M. Kataoka Y. . (2020). Bone marrow mononuclear cells activate angiogenesis via gap junction-mediated cell-cell interaction. Stroke 51, 1279–1289. doi: 10.1161/STROKEAHA.119.028072 32075549

[B32] Kim B. W. Lee J. S. Ko Y. G. (2019). Mycoplasma exploits mammalian tunneling nanotubes for cell-to-cell dissemination. BMB Rep. 52, 490–495. doi: 10.5483/BMBRep.2019.52.8.243 30673584 PMC6726209

[B33] Kumar N. M. Gilula N. B. (1996). The gap junction communication channel. Cell. 84, 381–388. doi: 10.1016/S0092-8674(00)81282-9 8608591

[B34] Labaied M. Jayabalasingham B. Bano N. Cha S.-J. Sandoval J. Guan G. . (2011). Plasmodium salvages cholesterol internalized by LDL and synthesized de novo in the liver. Cell. Microbiol. 13, 569–586. doi: 10.1111/j.1462-5822.2010.01555.x 21105984

[B35] Laird D. W. (1996). The life cycle of a connexin: gap junction formation, removal, and degradation. J. Bioenerg. Biomembr. 28, 311–318. doi: 10.1007/BF02110107 8844328

[B36] Lassen B. Ostergaard S. (2012). Estimation of the economical effects of Eimeria infections in Estonian dairy herds using a stochastic model. Prev. Vet. Med. 106, 258–265. doi: 10.1016/j.prevetmed.2012.04.005 22608299

[B37] Leighton B. Curi R. Hussein A. Newsholme E. A. (1987). Maximum activities of some key enzymes of glycolysis, glutaminolysis, Krebs cycle and fatty acid utilization in bovine pulmonary endothelial cells. FEBS Lett. 225, 93–96. doi: 10.1016/0014-5793(87)81137-7 3691808

[B38] Lin X. Wang W. Chang X. Chen C. Guo Z. Yu G. . (2024). ROS/mtROS promotes TNTs formation via the PI3K/AKT/mTOR pathway to protect against mitochondrial damages in glial cells induced by engineered nanomaterials. Part. Fibre Toxicol. 21, 1. doi: 10.1186/s12989-024-00562-0 38225661 PMC10789074

[B39] Liu K. Ji K. Guo L. Wu W. Lu H. Shan P. . (2014). Mesenchymal stem cells rescue injured endothelial cells in an *in vitro* ischemia–reperfusion model via tunneling nanotube like structure-mediated mitochondrial transfer. Microvasc. Res. 92, 10–18. doi: 10.1016/j.mvr.2014.01.008 24486322

[B40] Looker E. K. Aan F. J. Hatch C. J. Hughes C. C. W. Matter M. L. Fang J. S. (2023). Cx40 suppresses sprouting angiogenesis *in vitro*. Bioelectricity 5, 307–317. doi: 10.1089/bioe.2023.0034 40151625 PMC11949415

[B41] López-Osorio S. Silva L. M. R. Chaparro-Gutierréz J. J. Velásquez Z. D. Taubert A. Hermosilla C. (2020). Optimized excystation protocol for ruminant Eimeria bovis- and Eimeria arloingi-sporulated oocysts and first 3D holotomographic microscopy analysis of differing sporozoite egress. Parasitol. Int. 76, 102068. doi: 10.1016/j.parint.2020.102068 32006675

[B42] Luchetti F. Carloni S. Nasoni M. G. Reiter R. J. Balduini W. (2022). Tunneling nanotubes and mesenchymal stem cells: new insights into the role of melatonin in neuronal recovery. J. Pineal Res. 73, e12800. doi: 10.1111/jpi.12800 35419879 PMC9540876

[B43] Lv W. Li Z. Wang S. He J. Zhang L. (2024). A role for tunneling nanotubes in virus spread. Front. Microbiol. 15, 1356415. doi: 10.3389/fmicb.2024.1356415 38435698 PMC10904554

[B44] Mannell H. Kameritsch P. Beck H. Pfeifer A. Pohl U. Pogoda K. (2021). Cx43 promotes endothelial cell migration and angiogenesis via the tyrosine phosphatase SHP-2. Int. J. Mol. Sci. 23, 294. doi: 10.3390/ijms23010294 35008716 PMC8745637

[B45] Meens M. J. Alonso F. Le Gal L. Kwak B. R. Haefliger J.-A. (2015). Endothelial connexin37 and connexin40 participate in basal but not agonist-induced NO release. Cell. Commun. Signal. 13, 34. doi: 10.1186/s12964-015-0110-1 26198171 PMC4510910

[B46] Musil L. Cunningham B. Edelman G. Goodenough D. (1990). Differential phosphorylation of the gap junction protein connexin43 in junctional communication-competent and -deficient cell lines. J. Cell Biol. 111, 2077–2088. doi: 10.1083/jcb.111.5.2077 2172261 PMC2116332

[B47] Needs H. I. Glover E. Pereira G. C. Witt A. Hübner W. Dodding M. P. . (2024). Rescue of mitochondrial import failure by intercellular organellar transfer. Nat. Commun. 15, 988. doi: 10.1038/s41467-024-45283-2 38307874 PMC10837123

[B48] Norris R. P. Terasaki M. (2021). Gap junction internalization and processing *in vivo*: a 3D immuno-electron microscopy study. J. Cell Sci. 134, jcs252726. doi: 10.1242/jcs.252726 33277382

[B49] Odden A. Enemark H. L. Ruiz A. Robertson L. J. Ersdal C. Nes S. K. . (2018). Controlled efficacy trial confirming toltrazuril resistance in a field isolate of ovine Eimeria spp. Parasit Vectors 11, 394. doi: 10.1186/s13071-018-2976-4 29976240 PMC6034276

[B50] Ogawa Y. Akamatsu R. Fuchizaki A. Yasui K. Saino O. Tanaka M. . (2022). Gap junction–mediated transport of metabolites between stem cells and vascular endothelial cells. Cell Transplant. 31, 9636897221136151. doi: 10.1177/09636897221136151 36401520 PMC9679345

[B51] Okamoto T. Park E. J. Kawamoto E. Usuda H. Wada K. Taguchi A. . (2021). Endothelial connexin-integrin crosstalk in vascular inflammation. Biochim. Biophys. Acta (BBA) - Mol. Basis Dis. 1867, 166168. doi: 10.1016/j.bbadis.2021.166168 33991620

[B52] Scheckenbach K. E. L. Crespin S. Kwak B. R. Chanson M. (2011). Connexin channel-dependent signaling pathways in inflammation. J. Vasc. Res. 48, 91–103. doi: 10.1159/000316942 20926890

[B53] Silva L. M. R. Velásquez Z. D. López-Osorio S. Hermosilla C. Taubert A. (2022). Novel insights into sterol uptake and intracellular cholesterol trafficking during Eimeria bovis macromeront formation. doi: 10.3389/fcimb.2022.809606 PMC8878908 35223543

[B54] Stögerer T. Silva-Barrios S. Carmona-Pérez L. Swaminathan S. Mai L. T. Leroux L.-P. . (2023). Leishmania donovani exploits tunneling nanotubes for dissemination and propagation of B cell activation. Microbiol. Spectr. 11, e0509622. doi: 10.1128/spectrum.05096-22 37404188 PMC10434010

[B55] Taubert A. Silva L. M. R. Velásquez Z. D. Larrazabal C. Lütjohann D. Hermosilla C. (2018). Modulation of cholesterol-related sterols during Eimeria bovis macromeront formation and impact of selected oxysterols on parasite development. Mol. Biochem. Parasitol. 223, 1–12. doi: 10.1016/j.molbiopara.2018.06.002 29909067

[B56] Taubert A. Wimmers K. Ponsuksili S. Jimenez C. A. Zahner H. Hermosilla C. (2010). Microarray-based transcriptional profiling of Eimeria bovis-infected bovine endothelial host cells. Vet. Res. 41, 70. doi: 10.1051/vetres/2010041 20615380 PMC2920636

[B57] Taubert A. Zahner H. Hermosilla C. (2006). Dynamics of transcription of immunomodulatory genes in endothelial cells infected with different coccidian parasites. Vet. Parasitol. 142, 214–222. doi: 10.1016/j.vetpar.2006.07.021 16930845

[B58] Tishchenko A. Azorín D. D. Vidal-Brime L. Muñoz M. J. Arenas P. J. Pearce C. . (2020). Cx43 and associated cell signaling pathways regulate tunneling nanotubes in breast cancer cells. Cancers 12, 2798. doi: 10.3390/cancers12102798 33003486 PMC7601615

[B59] Van Rijen H. van Kempen M. J. Analbers L. J. Rook M. B. van Ginneken A. C. Gros D. . (1997). Gap junctions in human umbilical cord endothelial cells contain multiple connexins. Am. J. Physiol. 272, C117–C130. doi: 10.1152/ajpcell.1997.272.1.C117 9038818

[B60] Veenstra R. D. (1996). Size and selectivity of gap junction channels formed from different connexins. J. Bioenerg. Biomembr. 28, 327–337. doi: 10.1007/BF02110109 8844330

[B61] Vega J. L. Subiabre M. Figueroa F. Schalper K. A. Osorio L. González J. . (2013). Role of gap junctions and hemichannels in parasitic infections. BioMed. Res. Int. 2013, 589130. doi: 10.1155/2013/589130 24236292 PMC3819887

[B62] Velásquez Z. D. López-Osorio S. Mazurek S. Hermosilla C. Taubert A. (2021b). Eimeria bovis macromeront formation induces glycolytic responses and mitochondrial changes in primary host endothelial cells. Front. Cell. Infect. Microbiol. 11, 703413. doi: 10.3389/fcimb.2021.703413 34336724 PMC8319763

[B63] Velásquez Z. D. López-Osorio S. Waiger D. Manosalva C. Pervizaj-Oruqaj L. Herold S. . (2021a). Eimeria bovis infections induce G1 cell cycle arrest and a senescence-like phenotype in endothelial host cells. Parasitology 148, 341–353. doi: 10.1017/S0031182020002097 33100232 PMC7890351

[B64] Walters H. E. Cox L. S. (2021). Intercellular transfer of mitochondria between senescent cells through cytoskeleton-supported intercellular bridges requires mTOR and CDC42 signalling. Oxid. Med. Cell. Longev. 2021, 6697861. doi: 10.1155/2021/6697861 34373767 PMC8349290

[B65] Wang X. Veruki M. L. Bukoreshtliev N. V. Hartveit E. Gerdes H.-H. (2010). Animal cells connected by nanotubes can be electrically coupled through interposed gap-junction channels. Proc. Natl. Acad. Sci. U.S.A. 107, 17194–17199. doi: 10.1073/pnas.1006785107 20855598 PMC2951457

[B66] Wastling J. M. Xia D. Sohal A. Chaussepied M. Pain A. Langsley G. (2009). Proteomes and transcriptomes of the Apicomplexa--where’s the message? Int. J. Parasitol. 39, 135–143. doi: 10.1016/j.ijpara.2008.10.003 18996390

[B67] Willebrords J. Maes M. Crespo Yanguas S. Vinken M. (2017). Inhibitors of connexin and pannexin channels as potential therapeutics. Pharmacol. Ther. 180, 144–160. doi: 10.1016/j.pharmthera.2017.07.001 28720428 PMC5802387

[B68] Yasuda K. Park H.-C. Ratliff B. Addabbo F. Hatzopoulos A. K. Chander P. . (2010). Adriamycin nephropathy: a failure of endothelial progenitor cell-induced repair. Am. J. Pathol. 176, 1685–1695. doi: 10.2353/ajpath.2010.091071 20167859 PMC2843460

[B69] Yoo H. C. Yu Y. C. Sung Y. Han J. M. (2020). Glutamine reliance in cell metabolism. Exp. Mol. Med. 52, 1496–1516. doi: 10.1038/s12276-020-00504-8 32943735 PMC8080614

[B70] Zhao Y. Gao R. Ma J. Cui Y. Li J. Lin H. (2024). Characteristics of tunneling nanotube-like structures formed by human dermal microvascular pericytes *in vitro*. Tissue Cell. 89, 102431. doi: 10.1016/j.tice.2024.102431 38870572

