# Supplementary material for: Eimeria bovis-infected host cell-bystander cell interactions are essential for effective macromeront formation
Source: Front Cell Infect Microbiol. 2026 Aug 18;16:1915455. doi: 10.3389/fcimb.2026.1915455 (PMC13529501; doi:10.3389/fcimb.2026.1915455)
Supplement: Supplementary file 1 [file DataSheet1.pdf]

Antibodies / Dyes	Company	RRID	Cat. Number	Origin	Dilution
**Primary antibodies**					
Anti-Cx37	Invitrogen	AB_2533524	404200	Rabbit	1:85 (IF), 1:200 (WB)
Anti-Cx40	Invitrogen	AB_2533346	364900	Rabbit	1:85 (IF), 1:200 (WB)
Anti-Cx43	Cell Signaling	AB_2940791	83649	Rabbit	1:200 (IF), 1:500 (WB)
**Secondary antibodies**					
AlexaFluor 488	ThermoFisher	AB_143165	A11008	Goat	1:400
**Dyes**					
MitoView™ Green	Biotium™	–	70054	–	100 nM
DAPI Fluormount-G™	Invitrogen™	–	495952	–	1 drop / coverslip
