# Supplementary material for: Eimeria bovis-infected host cell-bystander cell interactions are essential for effective macromeront formation
Source: Front Cell Infect Microbiol. 2026 Aug 18;16:1915455. doi: 10.3389/fcimb.2026.1915455 (PMC13529501; doi:10.3389/fcimb.2026.1915455)
Supplement: Supplementary file 2 [file DataSheet2.pdf]

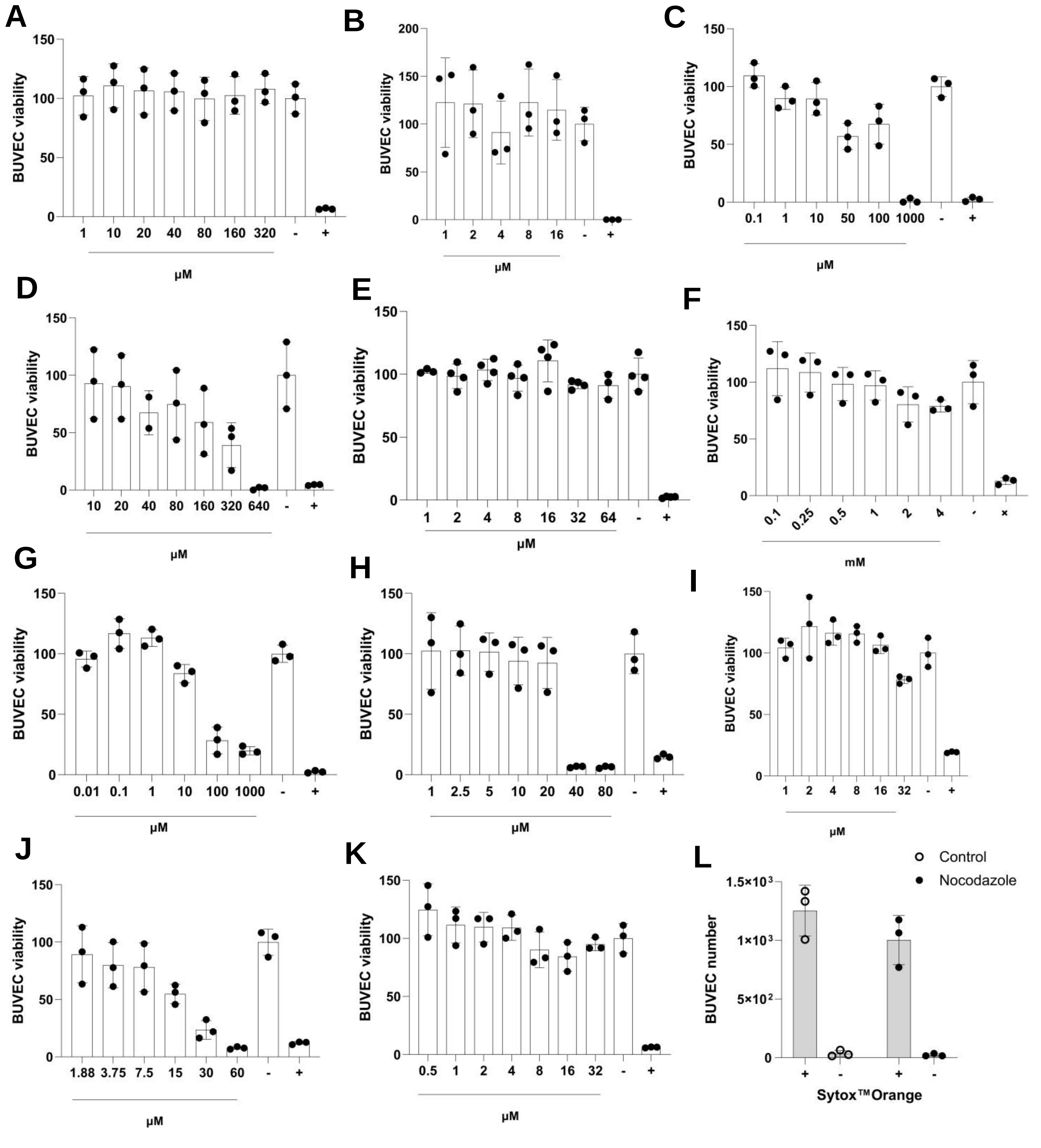

Suppl. Fig. 1. Assessment of cell toxicity. Cells were treated for 24 h with (A) arachidonic acid (B) N-acetylcysteine (C) carbenoxolone (D) Gap19 (E) Gap26 (F) fluordeoxyglucose(^{18}F) (G) rotenone (H) CL976 (I) ethylfluoracetate (J) U18666A or (K) 6-Diazo-5-oxo-L-norleucine. Cell toxicity was assessed by XTT tests. (L) Effects of nocodazole treatments on BUVEC. Quantification of viable (+) and dead (-) (Sytox™Orange) cells of nocodazole (50 nM) treated BUVEC after 24 h.

Suppl. Fig. 1.

Viability assays

Cell toxicity of the chemical compounds arachidonic acid, N-acetylcysteine, carbenoxolone, Gap19, Gap26, ^{18}F-fluordeoxyglucose, rotenone, CL976, ethylfluoracetate, U18666A, 6-Diazo-5-oxo-L-norleucine was evaluated using a colorimetric XTT assay (Madison, WI, USA) according to the manufacturer's instructions. Briefly, 1×10^5 BUVECs per well (n = 3) were cultured in 96-well plates and treated with the chemical compounds in a total volume of 50 µL for 24 h at 37 °C in a 5 % CO_2 atmosphere. Thereafter, 50 µL of XTT working solution were added per well and samples were incubated for additional 4 - 6 h at 37 °C in a 5 % CO_2 atmosphere. The resulting formazan products were quantified by measuring the optical density at 450 nm and 620 nm using a Varioskan Flash Multimode Reader (Thermo Fisher Scientific, Waltham, MA, USA). Plain medium-treated BUVECs served as negative controls, BUVECs treated with Triton X-100 (50 % v/v) were used as positive controls, and plain modECGM medium was used as blank for normalization (Fig. 1. A – K). Cell toxicity of the chemical compound nocodazole was tested by using Sytox™Orange (Thermo Fisher Scientific, Waltham, MA, USA) staining. Therefore, BUVEC were treated with nocodazole for 24 h (50 nM). Thereafter, BUVEC were stained by Sytox™Orange (5 µM, 5 min, RT), which only penetrates dying or dead cells. For negative and positive controls, non-treated and Triton X-treated BUVEC were used, respectively. The percentage of viable cells was calculated in randomly taken microscopic images (Fig. 1 A-B). Moreover, BUVEC treated with nocodazole exhibited no cell death (> 99.9 % of BUVEC viable) after 24 h (Fig. 1 L).

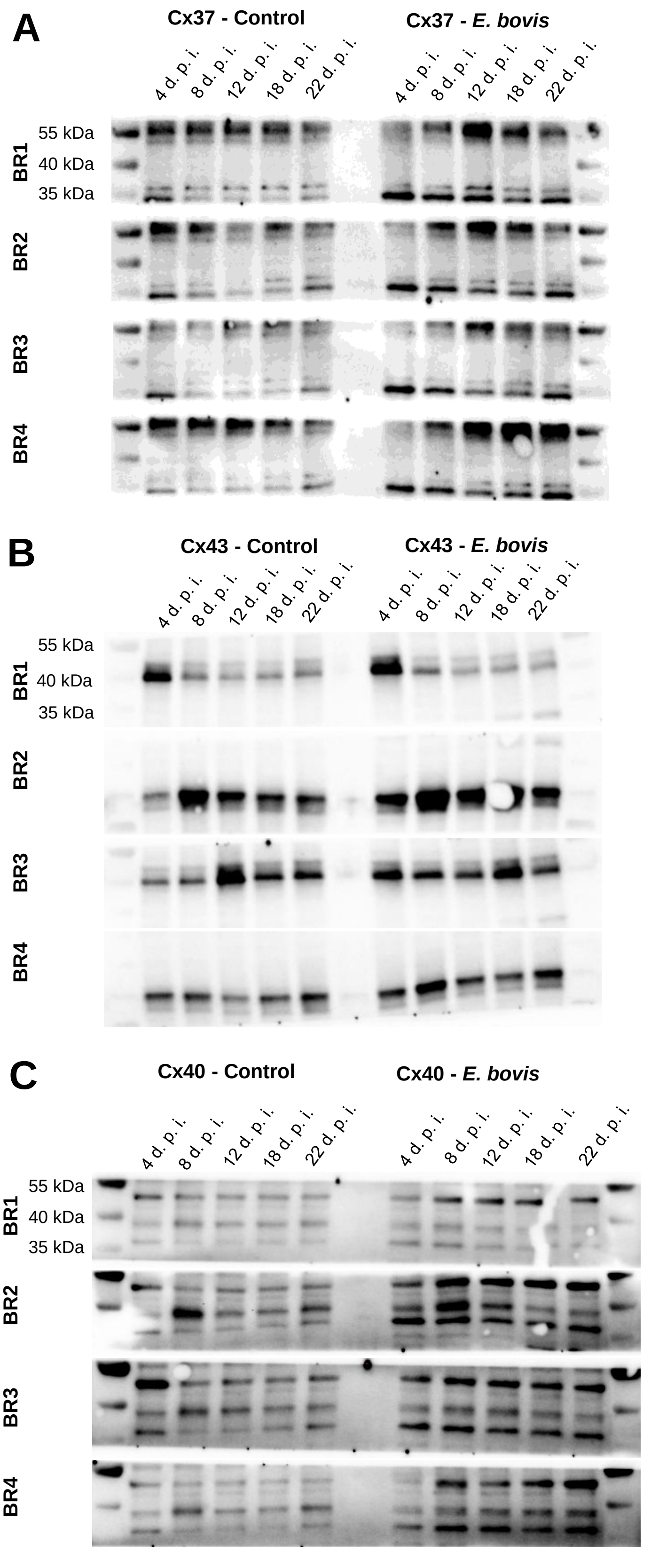

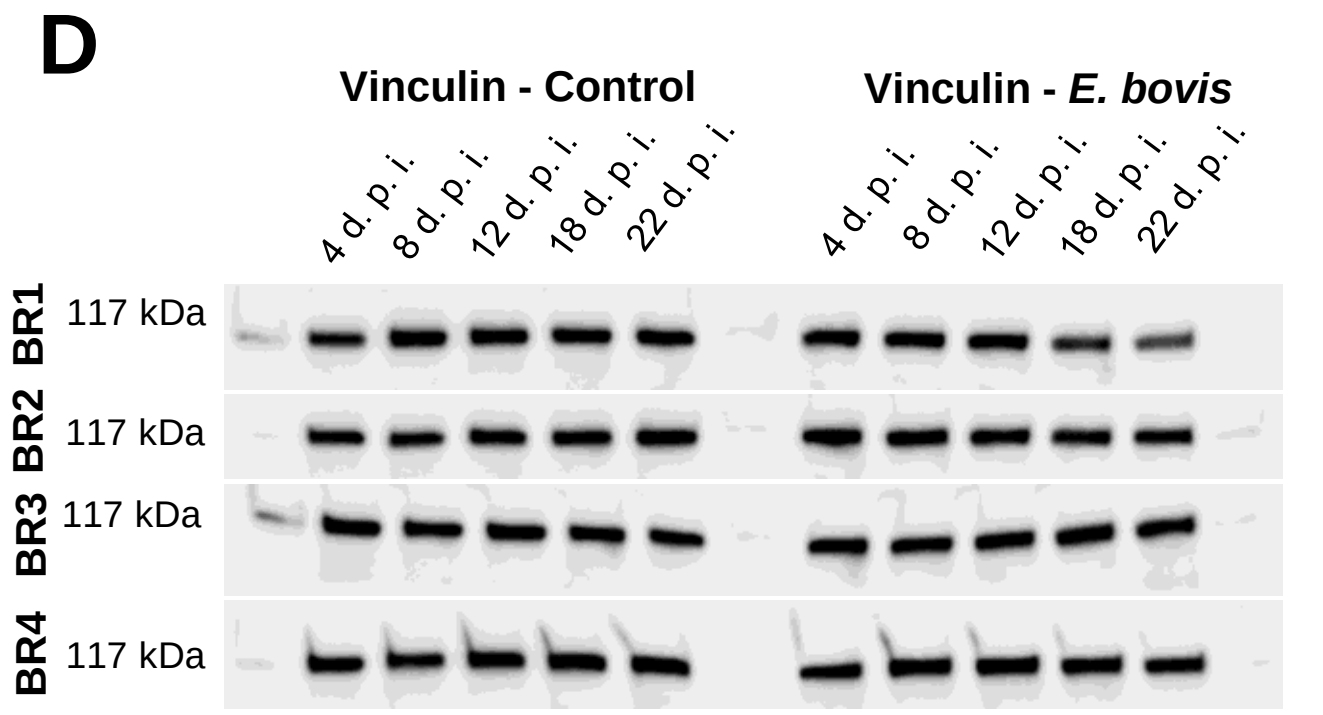

Suppl. Fig. 2. Expression of the connexins Cx37 (A), Cx40 (B) and Cx43 (C) throughout *E. bovis* merogony I detected by Western blotting. (D) Western blot of vinculin as loading control.

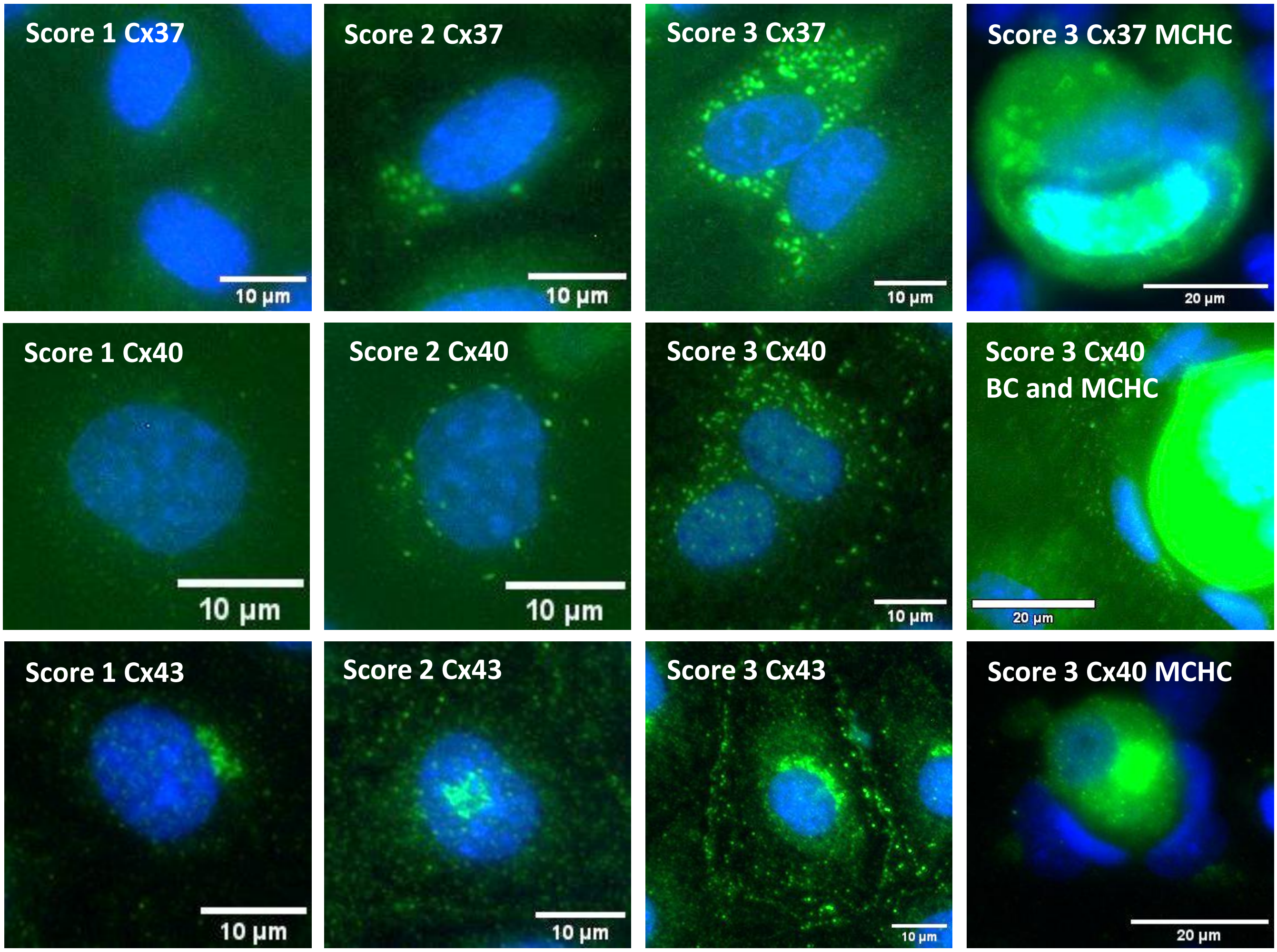

Suppl. Fig. 3. Illustration of the semi quantitative scoring strategy for connexin assessment in non-infected BUVEC, bystander cells (BC) and macromeront-carrying host cells (MCHCs)

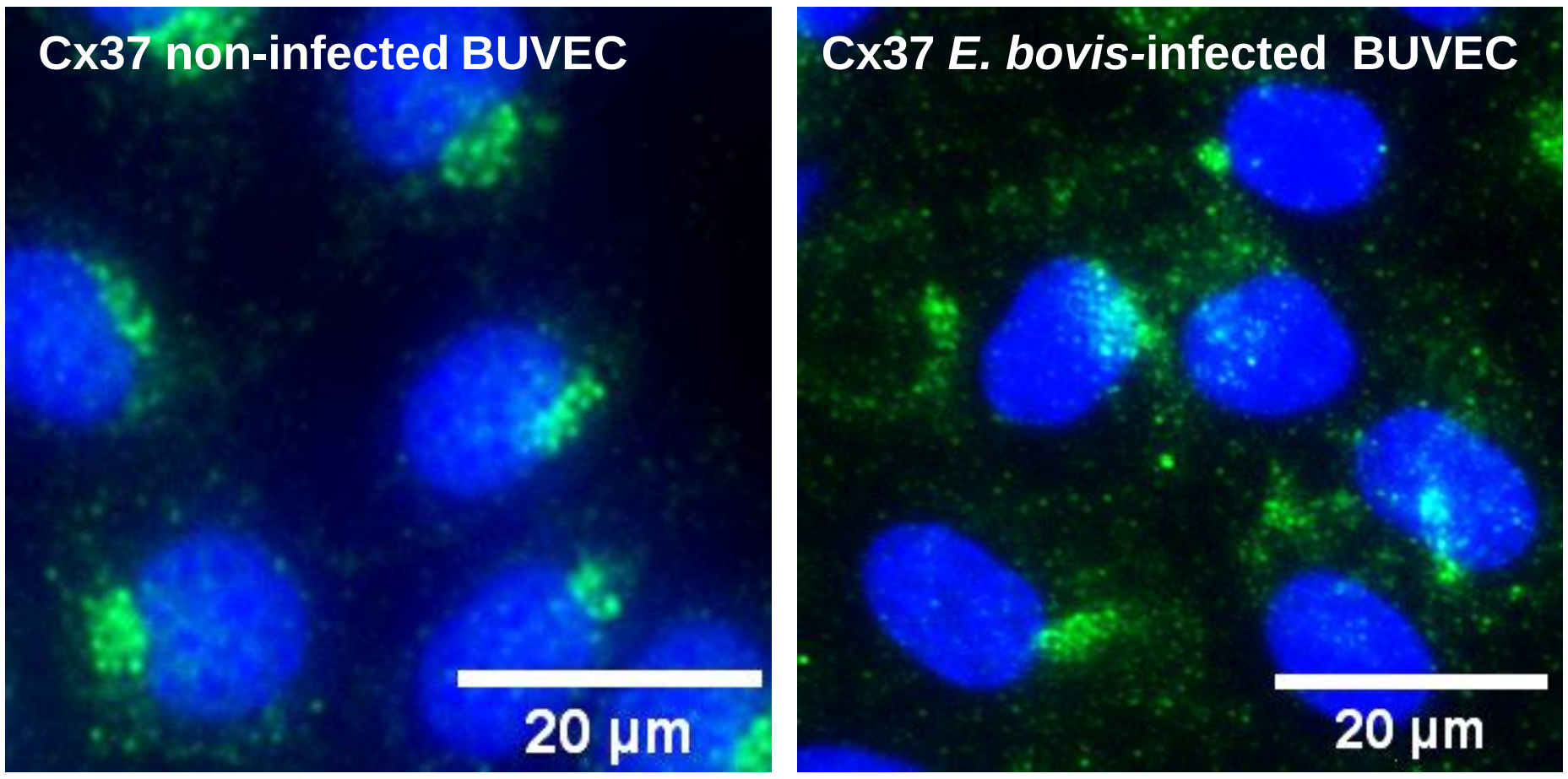

Suppl. Fig. 4. Others phenotype of Cx37 distribution in *E. bovis*-infected and non-infected BUVEC layers.

Suppl. Fig. 5 3D-volume rendering videos of *E. bovis*-infected BUVEC at 18 days p. i. stained for CX37 (Video 1), Cx40 (Video 2) and Cx43 (Video 3).

Movie files: https://next.hessenbox.de/index.php/s/MsgReJoHXYtzoMK
